# Protein lipidation in the tumor microenvironment: enzymology, signaling pathways, and therapeutics

**DOI:** 10.1186/s12943-025-02309-7

**Published:** 2025-05-07

**Authors:** Mengke Xu, Bo Xu

**Affiliations:** Chongqing Key Laboratory of Intelligent Oncology for Breast Cancer, Intelligent Oncology Innovation Center Designated by the Ministry of Education, Chongqing University Cancer Hospital and Chongqing University School of Medicine, Chongqing, 400030 China

**Keywords:** Lipidation, *N-*myristoylation, *S-*palmitoylation, *S-*prenylation, Tumor microenvironment

## Abstract

Protein lipidation is a pivotal post-translational modification that increases protein hydrophobicity and influences their function, localization, and interaction network. Emerging evidence has shown significant roles of lipidation in the tumor microenvironment (TME). However, a comprehensive review of this topic is lacking. In this review, we present an integrated and in-depth literature review of protein lipidation in the context of the TME. Specifically, we focus on three major lipidation modifications: *S-*prenylation, *S-*palmitoylation, and *N-*myristoylation. We emphasize how these modifications affect oncogenic signaling pathways and the complex interplay between tumor cells and the surrounding stromal and immune cells. Furthermore, we explore the therapeutic potential of targeting lipidation mechanisms in cancer treatment and discuss prospects for developing novel anticancer strategies that disrupt lipidation-dependent signaling pathways. By bridging protein lipidation with the dynamics of the TME, our review provides novel insights into the complex relationship between them that drives tumor initiation and progression.

## Introduction

**Fig. 1:**
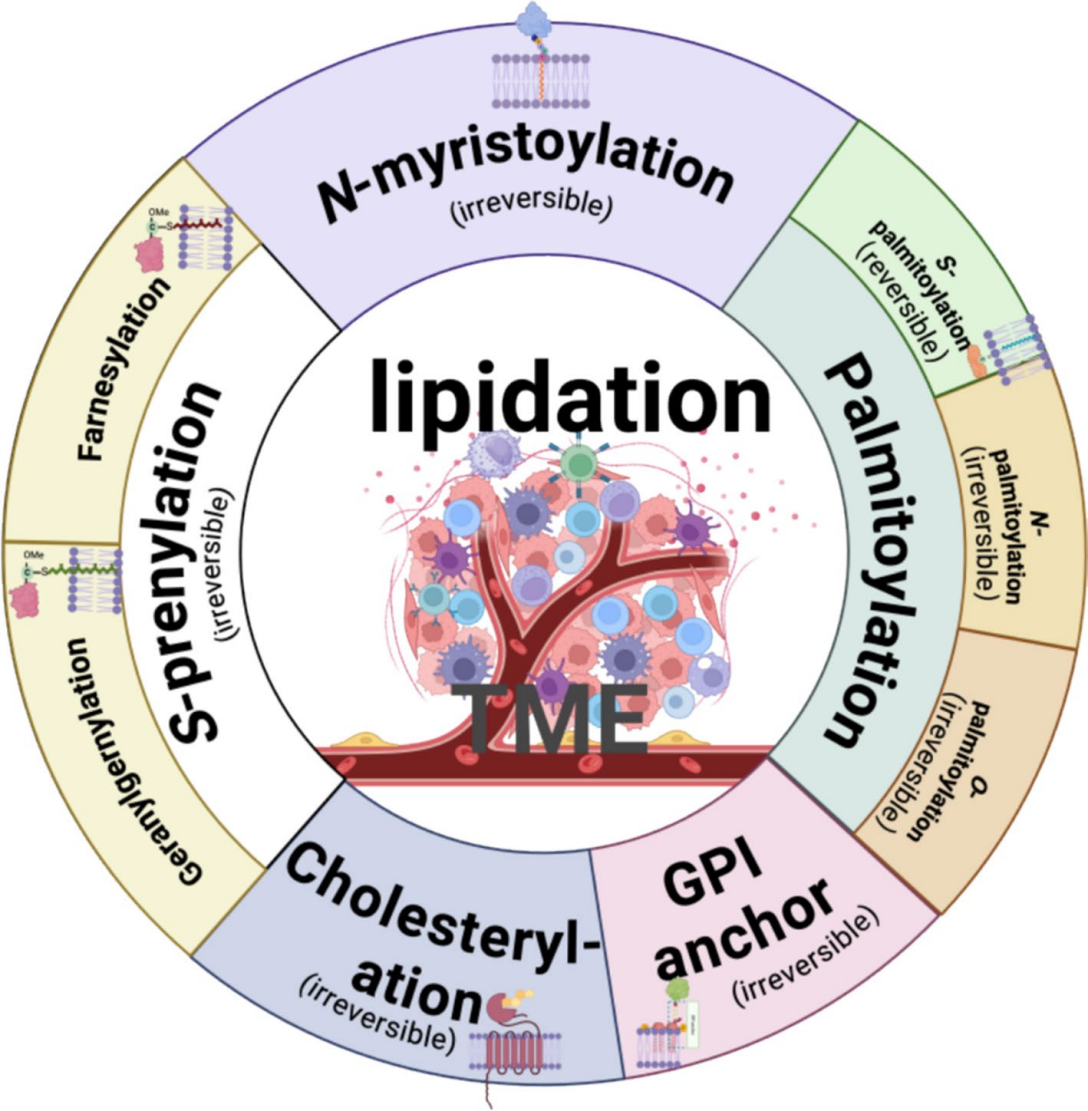
Overview of protein lipidation. Shown are five major types of lipidation modification, including *N-*myristoylation, *S-*palmitoylation, *S-*prenylation, Cholesterylation, GPI anchor, as well as their subtypes. GPI anchor: Glycosylphosphatidylinositol anchor; TME: Tumor microenvironment

**Fig. 2:**
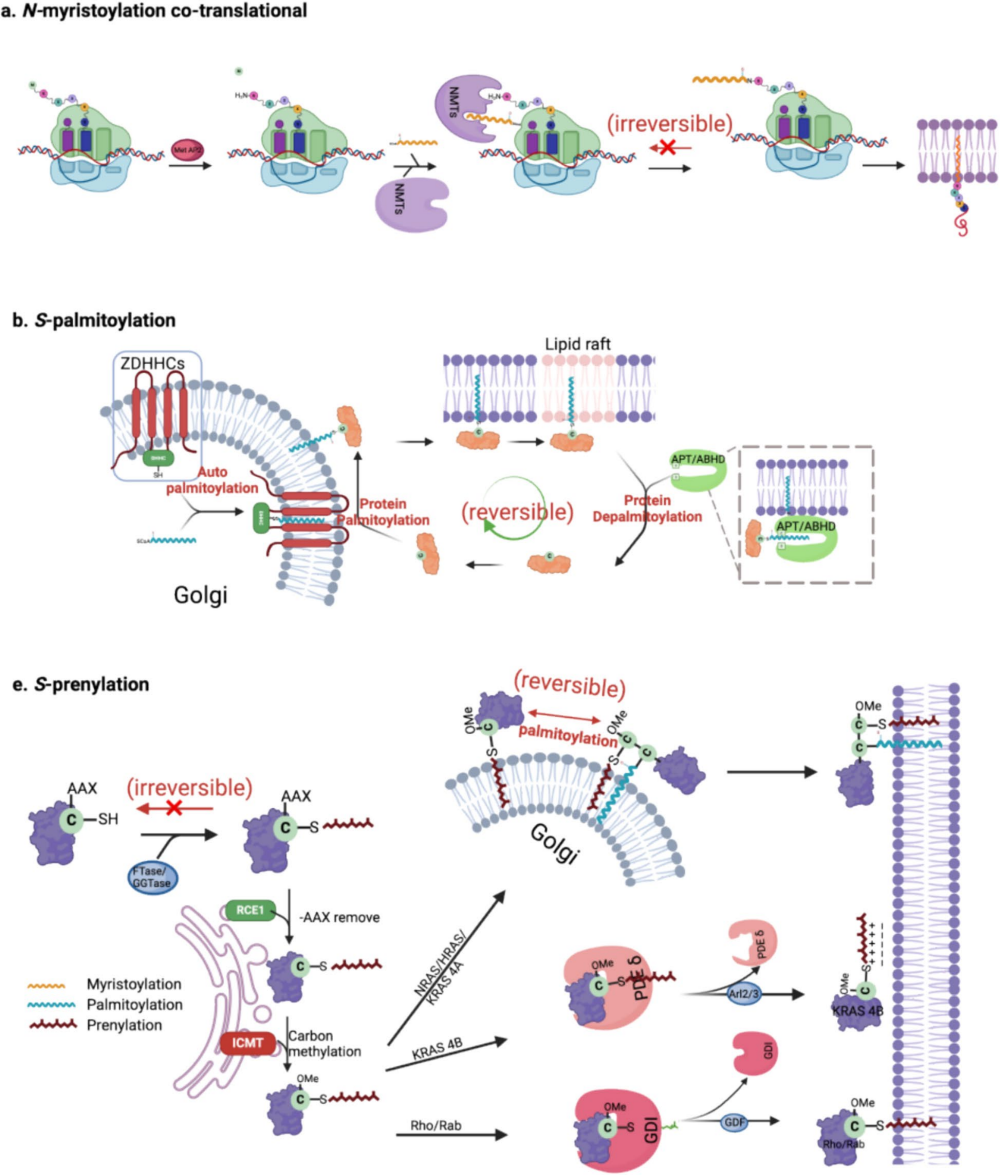
Processes of the three major classes for protein lipidation.** a** *N-*myristoylation is a co-translational lipid modification involving the irreversible covalent attachment of a myristoyl group (a 14-carbon saturated fatty acid derived from myristoyl-CoA) onto the N-terminal glycine residue of target proteins. During translation, the initiator methionine is typically removed by Methionine aminopeptidase 2 (MetAP2), exposing a glycine suitable for lipidation. This reaction is catalyzed by two closely related enzymes, *N-*myristoyltransferase 1 and 2 (NMT1/2), which display high catalytic efficiency (k_cat/K_M ~ 1.33 × 10^4 M⁻^1^ s⁻^1^), depending on the substrate peptide sequence context. Mechanistically, upon substrate binding, NMT1/2 enzymes undergo conformational transitions from an open, ligand-free state to a closed, catalytically competent state, precisely aligning peptide substrates and myristoyl-CoA for effective acyl transfer. The resultant irreversible lipid modification ensures stable membrane anchoring, thereby critically regulating protein trafficking, localization, and signaling pathways. **b** *S-*palmitoylation is a reversible, post-translational modification that typically occurs at the Golgi apparatus through a two-step enzymatic process. Initially, zinc finger DHHC-type palmitoyltransferases (ZDHHCs) undergo auto-palmitoylation, covalently attaching a 16-carbon palmitate derived from palmitoyl-CoA to their own cysteine residue. Subsequently, this palmitate moiety is transferred onto specific cysteine residues of substrate proteins. ZDHHC family members, such as ZDHHC20, exhibit high catalytic efficiency (k_cat/K_M ~ 1.13 × 10^5 M⁻^1^ s⁻^1^), facilitating rapid and selective protein *S-*palmitoylation. *S-*palmitoylated proteins typically display an enhanced affinity for plasma membrane localization, particularly within lipid rafts, influencing their trafficking, membrane partitioning, and downstream signaling dynamics. The reversible nature of this modification is mediated by depalmitoylation enzymes, predominantly acyl-protein thioesterases (APTs) and α/β hydrolase domain-containing proteins (ABHDs). These enzymes remove the palmitoyl group, enabling dynamic and reversible cycling of substrate proteins between intracellular compartments and the plasma membrane, thus fine-tuning protein function in response to cellular cues. **c** *S-*prenylation is a form of irreversible lipid modification initiated by the enzymatic attachment of either a farnesyl (15-carbon) or geranylgeranyl (20-carbon) isoprenoid lipid group to cysteine residues located within the conserved C-terminal CAAX motif (where C is cysteine, A an aliphatic amino acid, X determines prenylation specificity). Farnesyltransferase (FTase, catalytic efficiency k_cat/K_M ~ 1.8 × 10^5 M⁻^1^ s⁻^1^) and geranylgeranyltransferase (GGTase) catalyze these reactions, utilizing prenyl donors derived from intermediates of the mevalonate (MVA) pathway, farnesyl pyrophosphate (FPP) and geranylgeranyl pyrophosphate (GGPP), respectively. Prenylation markedly increases protein hydrophobicity, facilitating initial association with the cytosolic leaflet of the endoplasmic reticulum (ER). Subsequent post-prenylation modifications occur at the ER membrane, involving two sequential enzymatic steps to further enhance membrane affinity: Ras-converting enzyme 1 (Rce1) cleaves off the terminal -AAX amino acid residues. Isoprenylcysteine carboxyl methyltransferase (ICMT) then carboxymethylates the newly exposed C-terminal prenylcysteine residue. Different prenylated proteins exhibit distinct membrane-targeting strategies. NRAS and HRAS undergo additional *S-*palmitoylation by ZDHHC, which stabilizes their plasma membrane association within lipid rafts. In contrast, KRAS4B membrane localization primarily depends on electrostatic interactions facilitated by Phosphodiesterase δ (PDEδ) shuttle proteins. Meanwhile, prenylated small GTPases of the Rho and Rab families initially bind to guanine nucleotide dissociation inhibitors (GDIs) in the cytosol, before their transfer to the plasma membrane, regulating membrane cycling and signaling dynamics. ABHD: α/β-Hydrolase domain-containing protein; APT: Acyl-protein thioesterase; ARL2/3: ADP-ribosylation factor-like protein 2/3; FTase: Farnesyltransferase; GDI: Guanine nucleotide dissociation inhibitor; GDF: Guanine nucleotide dissociation factor; GGTase: Geranylgeranyltransferase; ICMT: Isoprenylcysteine carboxyl methyltransferase; NMT: *N-*myristoyltransferase; PDEδ:Phosphodiesterase δ; RAB: Ras-related in brain protein; RCE1: Ras-converting enzyme 1; RAS: Rat sarcoma viral oncogene homolog; RHO: Ras homolog family member; ZDHHC: Zinc finger DHHC-type containing protein

The tumor microenvironment (TME) encompasses a complicated network of dynamic interactions among tumor cells, stromal cells, vasculature, immune cells, extracellular matrix, and secreted factors [1]. Tumor initiation and progression are influenced not only by the intrinsic properties of tumor cells, but also by the multifaceted interactions within the TME that continuously shape tumor behavior [2]. Recent studies have shown that post-translational modifications (PTMs), particularly protein lipidation, play crucial roles in TME functions [3, 4]. Lipidation is the covalent attachment of lipid groups to proteins that increases hydrophobicity, facilitating membrane interactions that influence protein localization, signal transduction, and cellular interactions [5]. It includes *S-*prenylation, *N-*myristoylation, *S-*palmitoylation, and other less common types, such as *O*-palmitoylation, *N-*palmitoylation, C-terminal cholesterol esterification, and GPI anchor attachment (Fig. 1). This review focuses on three major lipidation modifications: *S-*prenylation, *S-*palmitoylation, and *N-*myristoylation. *N-*myristoylation is an irreversible lipidation involving the attachment of the 14-carbon fatty acid myristate to the N-terminal glycine of a substrate, catalyzed by *N-*myristoyltransferase (NMT) (Fig. 2a) [6, 7]. This modification typically occurs cotranslationally. During the protein synthesis, the initiator methionine is often removed by the methionine aminopeptidase 2(MetAP2), exposing a glycine residue at the N-terminus. The NMT then transfers the myristoyl group from myristoyl-coenzyme A (CoA) to this glycine residue [8]. In contrast, *S-*palmitoylation is a reversible modification that involves the attachment of palmitate, a 16-carbon saturated fatty acid (palmitate), to cysteine residues via a thioester bond, catalyzed by zinc-finger and aspartate–histidine–histidine–cysteine (ZDHHC) type (ZDHHC1 - 24, without ZDHHC10) family proteins. *S-*palmitoylation occurs in two steps: first, the ZDHHC enzymes autopalmitoylate at the DHHC cysteine residue, and then the palmitate is transferred to the cysteine residue of the target protein [9] (Fig. 2b). *S-*prenylation, also irreversible [10], refers to the covalent attachment of either a farnesyl (15-carbon) or, more commonly, a geranylgeranyl (20-carbon) group to the C-terminal cysteine residues, which must be part of a C-terminal cysteine-aliphatic-aliphatic-X (CAAX) box motif, of proteins, a reaction mediated by farnesyltransferase (FTase) and geranylgeranyltransferases I, II, and III (GGTase) [11, 12] (Fig. 2c). More importantly, many proteins undergo dual lipid modifications [13]; dual lipidation can occur in two scenarios: *N-*myristoylation with *S-*palmitoylation and *S-*prenylation with *S-*palmitoylation. The mechanism is two irreversible modifications occurring initially, followed by *S-*palmitoylation. The hydrophobicity conferred by the first modification is insufficient for precise localization, whereas the subsequent *S-*palmitoylation ensures accurate protein targeting.

Recent discoveries have shown that inhibitors targeting lipidation-related enzymes exhibit potent antitumor properties [14]. These inhibitors interfere with oncogenic signal transmission and cellular survival pathways, causing metabolic dysregulation [15, 16], cell cycle arrest [17], and programmed cell death [18–20]. They also increase the sensitivity of tumor cells to immune-driven attacks [21]. Several related drugs have been researched in the clinic [22–24].

Although some aspects of lipidation modifications in the TME have been revealed, no review has been published thus far to summarize the detailed interactions between them. Our review discusses recent progress in protein lipidation, focusing on *N-*myristoylation, *S-*palmitoylation, and *S-*prenylation. Our review synthesizes all relevant studies on lipidation in tumors and the TME, comprehensively elucidating the complex and critical regulatory interactions of lipidation within the TME. We provide an overview of current lipidation-related therapeutics in cancer treatment. Through this work, we aim to offer novel insights and recommendations for understanding disease mechanisms and advancing therapeutic strategies in oncology.

## Molecular mechanisms of lipidation

### Mechanisms of lipidation

#### *N-*myristoylation

*N*-myristoylation, catalyzed by NMT1/NMT2 [25], attaches a myristoyl group to Gly2 of nascent proteins. Despite shared catalytic folds (GNAT domains) and overlapping substrate specificity, NMT1 exhibits broader substrate range [26] and is overexpressed in cancers [27], linking it to tumorigenesis and embryonic development [28, 29] (Fig. 2a).

Substrate recognition depends on a conserved binding cleft: the N-terminal glycine (Gly2) and adjacent residues (e.g., Cys3) are anchored via hydrophobic pockets and electrostatic interactions (e.g., Asp183/185/471), while Myr-CoA binding triggers conformational closure to form a stable ternary complex. Competition with NatA in ribosomal exit tunnels dynamically regulates protein acetylation versus *N-*myristoylation, expanding functional diversity. Evolutionarily, the N-terminal pentapeptide (residues 2–6) encodes a dual-modification "code", where Cys3 - 5 positions enable sequential *N-*myristoylation-palmitoylation synergism [26]. Clinically, *N-*myristoylation stabilizes oncoproteins (e.g., Src kinases) and modulates immune responses, making NMT1 a therapeutic target in TME [8, 30, 31].

#### *S-*palmitoylation

ZDHHC family enzymes catalyze *S-*palmitoylation through their conserved DHHC-cysteine-rich structural domains and at least four transmembrane structural domains [32], whose crystal structures (e.g., human ZDHHC20 and zebrafish ZDHHC15) show that the transmembrane structural domains form a hydrophobic lumen that selects for the length of the acyl chain (C14:0 to C22:0, usually C16:0) through residues in the cavity [33, 34]. Catalysis follows a two-step ping-pong mechanism: first, the conserved Cys of the DHHC motif reacts with palmitoyl-CoA to generate an acyl-enzyme intermediate (auto-*S*-palmitoylation); subsequently, the acyl group is transferred to a near-membrane Cys residue of the substrate protein.The substrate selectivity of the ZDHHC enzyme is determined by the subcellular localization because it dictates where enzymes and substrates can interact, the specific structural domains (such as the anchor protein repeat motif of ZDHHC13/17 [35] or PDZ-binding motif of ZDHHC5 [36]) and substrate sequence features (e.g., N-terminal *N-*myristoylation sequence of near-membrane Cys) synergistically [37]. The Golgi membrane is its primary active site [38], This modification dynamically regulates protein-membrane interactions by enriching targets (e.g., Fas, death receptor 4 (DR4)) in lipid rafts [39, 40], maintaining Golgi-PM distribution [41], and mediating lysosomal sorting via TMEM55B-dynein complexes [42].

The reversibility of this modification is mediated by acyl protein thioesterases (APT1/2, PPT1) and α/β hydrolases (ABHD10, ABHD17 A-C) [37], which enable the protein to switch palmitoylated/de-palmitoylated states on a second to hourly scale [43], dynamically modulating membrane affinity, localization, stability, and signal transduction functions [44].The results demonstrate the spatiotemporal plasticity of lipid modification in cellular response (Fig. 2b).

#### *S-*prenylation

*S-*prenylation involves the attachment of a farnesyl or geranylgeranyl group to cysteine residues within a CAAX motif, which determines the type of *S-*prenylation [45]. FTase catalyzes farnesylation, while GGTase mediates geranylgeranylation. GGTase exists in three isoforms; FTase and GGTase I share an α subunit but differ in β subunits for distinct substrate specificities [46]. Differential electrostatic potentials on their β subunits dictate substrate preference: FTase's β subunit favors serine/methionine at the "X" position of CAAX motif, whereas GGTase-I prefers leucine, these enzymes do not manifest mutually exclusive substrate specificity [47], some proteins can be processed by GGTase-I when FTase is inhibited [45, 48, 49]. A reasonable explanation is FTase favors Farnesyl pyrophosphate (FPP, 15 carbons) because its shorter chain positions the diphosphate closer to the catalytic Zn^2^⁺, while GGTase-I prefers Geranylgeranyl pyrophosphate (GGPP 20 carbons) due to deeper insertion into its hydrophobic pocket. FPP binds weakly to GGTase-I (330-fold lower affinity than GGPP), but its diphosphate still partially aligns with the active site, allowing limited catalysis [50]. GGTase-II specifically modifies Rab proteins with double-cysteine motifs for dual geranylgeranylation, which is essential for vesicle transportation; the geranylgeranylation of Rab occurs through two distinct mechanisms. First, Rab escort proteins (REPs) initially bind to Rab, facilitating its subsequent interaction with GGTase-II. In the second mechanism, REPs first associate with GGTase-II, and this complex then binds to Rab [51–57]. GGTase III modifies specific proteins, such as FBXL2 and Ykt6 [58, 59].

Indeed, *S-*prenylation involves three significant steps before membrane localization: preliminary *S-*prenylation, proteolytic cleavage, and carboxymethylation [60–62] (Fig. 2c). Initially, *S-*prenylation transports proteins to the ER. Secondly, the AAX residues are cleaved from the CAAX motif of the protein by Ras converting enzyme 1 (Rce1), which makes the protein more receptive to membrane binding [63]. Finally, isoprenylcysteine carboxyl methyltransferase (ICMT) methylates prenylated cysteine, further neutralizing its negative charge and enhancing membrane affinity [64, 65]. The modified protein is then targeted to membranes and dynamically distributed among distinct membrane regions (PDE6δ) [66], guanine nucleotide dissociation inhibitors (GDIs) [67], and Smg GDS [68]. Other dissociation factors, such as Arl2 and Arl3 and GDI displacement factors [67], are said to release these proteins from solubilizing factors and relocate them to membranes. KRAS4B exemplifies a unique targeting mechanism, its PM localization depends on electrostatic interactions between the farnesyl moiety and anionic phospholipids [69], forming a farnesyl-electrostatic switch regulated by PKC [70] and Ca^2^⁺/calmodulin [71].

By no means being less necessary, *S-*prenylation has been shown to protect proteins against degradation due to enhanced folding and stability, a phenomenon described for Rab1B and YKT6 [72, 73]. Conversely, *S-*prenylation can also serve as degradation pathways for Rho proteins, thereby regulating protein turnover [74]. Returning to the subject, *S-*prenylation increases the affinity of proteins for interacting upstream or downstream signaling molecules, such as the interaction between KRas4B and hSOS1, which promotes GDP–GTP exchange and activates the Ras signaling cascade [75, 76].

#### Dual modification

**Fig. 3:**
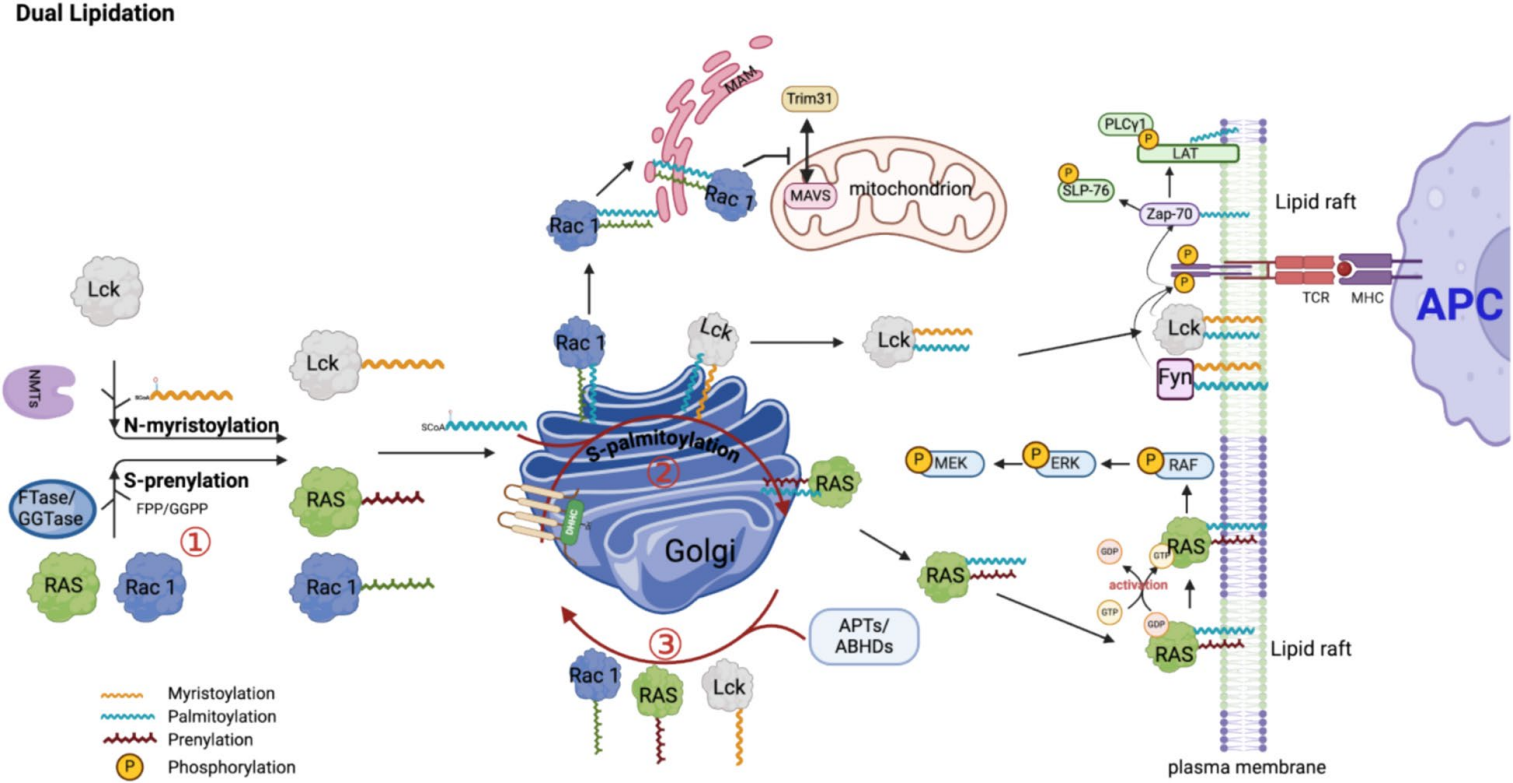
The comprehensive kinetic model illustrates dual lipidation interplay within the TME signaling pathways. A detailed representation of dual lipidation dynamics in T-cell receptor (TCR) signal transduction, mitochondrial antiviral signaling and Ras-dependent proliferative signaling within the TME. Initially, the proto-oncogene tyrosine-protein kinase Lck undergoes *N-*myristoylation. Subsequently, Lck undergoes reversible *S-*palmitoylation at cysteine residues, primarily catalyzed by ZDHHC within the Golgi apparatus. Palmitoylation significantly enhances Lck membrane affinity, promoting localization into lipid raft microdomains. Upon antigen presentation by antigen-presenting cells (APCs), dual lipidation anchors Lck at the plasma membrane, where it phosphorylates ITAM motifs on CD3 chains, initiating the T-cell activation cascade. Downstream signaling involves adaptor proteins (LAT, SLP- 76) whose *S*-palmitoylation stabilizes signaling assemblies within lipid rafts, enhancing the activity of PLCγ1, triggering secondary messenger production and signal amplification. A parallel dual lipidation mechanism governs small GTPases, notably HRAS and NRAS, involving initial irreversible *S-*prenylation catalyzed by FTase. This prenylation event primes Ras proteins for subsequent reversible *S-*palmitoylation via ZDHHC enzymes at the Golgi apparatus, enhancing their membrane microdomains: affinity and facilitating efficient trafficking to the lipid raft of plasma membrane (PM). At the PM, palmitoylated Ras isoforms upload GTP and tranlocated to the disordered (non-raft) regions of the PM then activate downstream effectors (RAF/MEK/ERK pathway), which promote oncogenic signaling cascades critical for proliferation and survival in the TME. *S*-palmitoylation dynamically modulates Ras localization between distinct cellular compartments. Thioesterase enzymes (APT1/2) reversibly remove the palmitoyl groups, enabling Ras isoforms to cycle between the PM and Golgi. This continuous palmitoylation–depalmitoylation cycling tightly regulates Ras signaling activity and membrane occupancy. The small GTPase Rac1 similarly undergoes dual lipidation cycles. Following viral infection, Rac1 translocates into cholesterol-enriched microdomains within mitochondria-associated membranes (MAMs), where it inhibits the interaction between MAVS and the E3 ligase Trim31. This prevents Trim31-mediated ubiquitination of MAVS, thereby blocking MAVS aggregation and downstream antiviral activation. ABHD: α/β-Hydrolase domain-containing protein; APC: Antigen-presenting cell; APT: Acyl-protein thioesterase; ERK: Extracellular signal-regulated kinase; Fyn: Proto-oncogene tyrosine-protein kinase Fyn; LAT: Linker for activation of T cells; Lck: Lymphocyte-specific protein tyrosine kinase; MAVS: Mitochondrial antiviral-signaling protein; MEK: Mitogen-activated protein kinase kinase; MHC: Major histocompatibility complex; PLCγ1: Phospholipase C gamma 1; Rac1: Ras-related C3 botulinum toxin substrate 1; RAF: Rapidly accelerated fibrosarcoma kinase; RAS: Rat sarcoma viral oncogene homolog; SLP- 76: SH2 domain-containing leukocyte protein of 76 kDa; TCR: T-cell receptor; Trim31: Tripartite motif-containing protein 31

These lipidations often act in concert (“dual lipidation”) to stably embed proteins at specific membrane microdomains. Notably, one lipid can “prime” the attachment of the second, for example, prenylation of Ras and *N-*myristoylation of Src-family kinases are prerequisite for their subsequent *S-*palmitoylation [77]. Src-family kinases (Lck, Fyn, Lyn, etc.), endothelial nitric oxide synthase (eNOS) [78–81], fibroblast growth factor receptor substrate (FRS2α) [82], LAMTOR1 [83], Gαi1 [13, 84, 85] are myristoylated at the N-terminus and then palmitoylated on nearby cysteines, a dual modification that tethers them to the lamellar structure of negatively charged phospholipids [84], lipid rafts [82], lysosome [83], or coated pits [80]. The sequential *S-*palmitoylation is evolutionarily encoded within the N-terminal pentapeptide (residues 2–6): proteins with Cys3 – 5 achieve ~ 75% *N-*myristoylation efficiency, of which 33–50% undergo *S-*palmitoylation due to additional Cys residues, reflecting a genetic imprinting strategy where weak myristoyl anchoring is reinforced by strong palmitoyl interactions [26] (Fig. 3) Table 1.

A parallel mechanism governs the dual modification of HRAS and NRAS, where *S-*prenylation is an obligatory first step that primes Ras for subsequent *S-*palmitoylation [86, 87]. Once palmitoylated, Ras is efficiently trafficked from the Golgi to the plasma membrane (PM), where it interacts with its regulators and effectors. Reversible removal of the palmitate by APT1/2 allows Ras to cycle back to the Golgi, maintaining a dynamic “on/off” localization loop. Kinetic studies have shown that the *S-*palmitoylation half-life of HRAS and NRAS ranges from approximately 30 min to 2 h [88], which is essential for fine-tuning their signaling outputs. In contrast, KRas4B lacks a *S-*palmitoylation site and instead relies on a polybasic tail, serving as an electrostatic membrane anchor [69], and the chaperone protein PDEδ to achieve stable PM association [89]. The presence or absence of *S-*palmitoylation also determines the specific membrane microdomains occupied by Ras isoforms. Palmitoylated HRAS and NRAS preferentially partition into order regions like lipid rafts and caveolae domains; but upon GTP loading it translocates out of rafts into the disordered (non-raft) regions of the PM, a shift critical for effective activation of downstream pathways like Raf/MEK/ERK [90]. If HRAS is artificially tethered in rafts continuously by adding an extra palmitate or preventing depalmitoylation, its ability to activate the Raf/MEK/ERK pathway is significantly impaired. Conversely, KRAS4B predominantly resides in non-raft regions regardless of its activation state, which affects its interaction with distinct effectors [90]. Thus, the dual lipidation of HRAS and NRAS provides a finely tuned mechanism for regulating signal amplitude and duration. Palmitoylation not only anchors Ras at the membrane to facilitate effective signal transduction but, owing to its reversible nature, also permits Ras to disengage, be recycled, or degraded—preventing overstimulation under normal physiological conditions.

By ensuring proper Ras localization and activation, dual lipidation facilitates crosstalk between Ras-driven oncogenic signals and the TME. Mutant Ras proteins are often locked in a sustained GTP-bound state, leading to continuous activation of the MAPK/ERK and PI3K-AKT pathways. This persistent signaling drives relentless cell proliferation, modulates immune responses by influencing the recruitment, activation, and differentiation of immune cells, and upregulates immune checkpoint molecules such as PD-L1 and CD47 [91].

### Methodology for lipidation

Methods for studying protein lipidation face several challenges, primarily due to the low abundance of lipidated proteins in cells, the diversity of lipid types and modification sites, and the lack of specific labeling and enrichment strategies, which increase the difficulty of lipidation research.

#### Traditional methods

Traditional methods for studying lipidation, such as radioactive isotope labeling with compounds like [3H]palmitic acid [92], [3H]myristate, and [I125]myristate [13] have been valuable for detecting lipidation target proteins. Although this technique provided valuable tools for early research, it has limitations, including safety concerns, long detection times, and complex procedures. This method is usually unsuitable for large-scale screening due to its sensitivity and measurement throughput limitations. For the *S-*palmitoylation, alternative proteomics approaches, including acyl-biotin exchange (ABE) [93] and acyl-resin assisted capture (acyl-RAC) [94], exploit the labile nature of the thioester bond in *S-*palmitoylation to identify thousands of *S-*palmitoylation proteins through selective chemical cleavage with hydroxylamine. However, these methods have limitations, including high background and the inability to determine the acyl chain length [95].

#### Click chemistry

With recent advancements in chemical biology, click chemistry has emerged as a powerful approach for PTM studies, primarily due to its high specificity, efficiency, and compatibility with biological systems [96, 97]. Unlike traditional methods relying on radioactive labeling or antibodies, click chemistry uses lipid probes modified with alkynes or azide groups, which react selectively and efficiently with complementary fluorophores, affinity tags, or drug carriers via bioorthogonal cycloaddition reactions. This has enabled detailed analyses in diverse applications, including cell imaging, flow cytometry, gel electrophoresis, and immunoprecipitation [98]. A novel approach for studying *N-*myristoylation by designing photocrosslinkable and clickable myristic acid analog probes, enabling the capture of *N-*myristoylation-specific protein–protein interactions in living cells without genetic modification [99]. However, copper-catalyzed based click chemistry faces several challenges. The introduction of azides or alkynes and copper ions, can potentially alter the natural biological activity and raises concerns regarding potential toxicity [100]. To address these limitations, researchers have developed copper-free bioorthogonal reactions, such as strain-promoted azide-alkyne cycloaddition (SPAAC) [101] and tetrazine ligation [102]. The reaction kinetics of SPAAC is slower than that of CuAAC, but its biocompatibility in living cells is unquestionable [103]. Tetrazine ligation, involving inverse electron-demand Diels–Alder (IEDDA) reactions between tetrazines and strained alkenes or alkynes, offers rapid reaction rates and excellent bioorthogonality, making it particularly suitable for in vivo applications [102]. However, these bioorthogonal cycloaddition reactions often use tensile olefins or alkynes as reactants, which are difficult to synthesize and have poor stability. Additionally, novel bioorthogonal cycloaddition reactions have been developed to simultaneously study multiple biomolecules in complex systems, providing new strategies for investigating lipidation processes [104].

#### Mass spectrometry

Liquid chromatography-tandem mass spectrometry (LC–MS/MS) and matrix-assisted laser desorption/ionization mass spectrometry imaging (MALDI-MSI) are pivotal techniques in the study of lipidation. LC–MS/MS integrates liquid chromatography with mass spectrometry to separate peptides and analyze their sequences, enabling the identification and quantification of lipidation [105]. Since lipidation is a low-abundance modification, enrichment strategies are essential for detection. Common approaches include click chemistry-based metabolic labeling, genetic encoding of unnatural amino acids (UAAs) with in vivo installation of palmitate mimics via the IEDDA reaction [106], ABE [107], and the Acyl-RAC all of which improve sensitivity in LC–MS/MS analysis and facilitate dynamic quantification of *S-*palmitoylation [108]. This method is highly sensitive and specific, making it indispensable in proteomics research. For example, LC–MS/MS has been employed to profile human protein *S-*palmitoylation [109], and our lab utilizes this technique to identify substrates of ZDHHC20 and map *S-*palmitoylation sites [110]. Notably, the click chemistry proteomics strategy takes advantage of the unique reversibility of *S-*palmitoylation, combined with pulse-chase methods to observe the reversible changes of protein modifications over time, revealing their dynamic regulatory mechanisms [111].

Conversely, MALDI-MSI allows for the in situ analysis of proteins and their modifications within tissue sections, preserving spatial context. By ionizing molecules directly from tissue surfaces, MALDI-MSI provides detailed maps of protein distributions and their PTMs across different tissue regions [112]. This technique has been applied to enhance the detection of extracellular matrix (ECM) proteins, which are challenging to analyze due to their size, insolubility, and extensive modifications like glycosylation. Innovative methods combining tissue decellularization with enzymatic digestion have improved ECM protein detection using MALDI-MSI, facilitating more accurate spatial mapping of these proteins [113]. For palmitoylated proteins, a major challenge is detecting the intact protein or peptide. A clever solution is on-tissue chemical derivatization [114]; for example, treating a tissue section with hydroxylamine cleaves thioester-linked palmitate, producing a predictable mass shift, which MALDI-MSI can then detect to generate a spatial map of palmitoylated protein abundance. MALDI-MSI can achieve a spatial resolution of 5–10 μm [115], but since it lacks chromatographic separation, coexisting biomolecules such as proteins, lipids, and inorganic salts can interfere with desorption and ionization efficiency. Therefore, optimized tissue preparation methods are required to improve detection accuracy [116]. LC–MS/MS excels in detailed characterization and quantification of PTMs in complex protein mixtures, while MALDI-MSI provides valuable insights into the spatial distribution of modified proteins within tissues.

Recent advancements in lipidation research have leveraged metabolic labeling, click chemistry, and nanoLC-MS/MS-based proteomics to achieve high-throughput, site-specific analysis of protein *S-*prenylation [117], this study introduces a novel approach utilizing alkyne-tagged isoprenoid analogs (YnF and YnGG) for bioorthogonal labeling of prenylated proteins in living cells, followed by selective enrichment via click chemistry. This strategy overcomes longstanding challenges in detecting lipid modifications, including hydrophobicity and poor ionization efficiency, which have historically hindered mass spectrometric analysis. The method demonstrates high sensitivity, as evidenced by the low nanomolar to micromolar IC₅₀ values observed for FTase inhibitors, highlighting its effectiveness in quantifying lipidation dynamics with precision. Furthermore, advanced quantification strategies such as stable isotope labeling by amino acids in cell culture (SILAC) [118, 119] or tandem mass tagging [120] improve accuracy and reproducibility.

#### Advanced imaging technologies

Unlike click chemistry, imaging techniques such as Förster Resonance Energy Transfer (FRET) allow real-time observation of molecular interactions within living cells, providing continuous, non-invasive monitoring of dynamic biological processes. While click chemistry primarily detects biomolecular events at specific time points, offering "snapshots" reflecting target molecule concentrations (with detection limits as low as 1.8–7.3 μg L⁻^1 ^[121], FRET focuses on the spatial dimension, detecting interactions within the 1–10 nm range [122]. Thus, FRET is highly sensitive for studying protein–protein interactions and visualizing biological processes such as T cell signals and the signal transduction [123] in the TME. Recently, FRET has been increasingly utilized in lipidation studies to investigate the distances and interactions among lipidated proteins by labeling them with donor and acceptor fluorophores [124]. One effective approach involves engineering reporter proteins that exhibit FRET signal changes upon lipidation. For instance, a fluorescent protein fused with a known lipidation-target sequence translocates to cellular membranes when lipidated, changing the proximity between donor and acceptor fluorophores [125, 126]. This allows dynamic, real-time observation of lipidation events within living cells, providing high sensitivity capable of detecting subtle structural and functional changes in proteins. Additionally, FRET spectroscopy can analyze fluorescence variations to infer the size, shape, and composition of protein complexes, offering insights comparable to mass spectrometry [127]. However, this technology still faces challenges, such as the lengthy exploration of fluorescence donors and acceptors, as well as the need to carefully consider the spectral overlap between the donor and acceptor, which demands high experimental precision.

With the continuous advancement of imaging technologies, fluorescently labeled multifunctional peptides have also been used to study lipid-modified proteins. For example, multifunctional peptides labeled with fluorescent tags based on the C-terminus of naturally isoprenylated protein cell division control protein (CDC42) [128] or by introducing ketone into myristoylated ADP-ribosylation factor 1 (ARF1) proteins followed by fluorescence labeling with hydrazine conjugated to aminobenzene azides, allow the modified proteins to be detected by fluorescence microscopy [129], enabling localization and dynamic observation of these proteins within living cells.

Moreover, super-resolution microscopy has provided a new perspective on the visualization of lipid modifications. For instance, combining a novel bioorthogonal cholesterol probe (chol-N₃) with super-resolution microscopy allows for direct visualization and characterization of lipid rafts in living cells with unprecedented resolution [130]. This approach, which combines bioorthogonal chemistry and super-resolution microscopy, offers new possibilities for dynamic lipidation observations, and it is expected that this method will be widely applied in lipid modification research in the future. Significant advances have recently been made in super-resolution stimulated Raman scattering (SRS) microscopy. The development of the Adam point-cloud deconvolution algorithm has improved the spatial resolution of SRS microscopy, enabling the reconstruction of high-resolution images from SRS data using deep learning techniques. This makes it possible to conduct nanoscale label-free imaging and study metabolic dynamics in living cells [131], which provides a more precise and in-depth tool for studying the dynamic processes of lipidation.

#### Multi-omics integration

With more profound studies of multi-omics and the development of multi-dimensional data such as spatial transcriptomics, researchers have been able to delve deeper into the dynamics of cells in space and time [132]. The growing recognition of biological interconnectedness has driven the integration of various omics methods (e.g., proteomics, metabolomics, and lipidomics) to provide a more comprehensive view of lipidation networks. Informatics tools have significantly supported this integration, with platforms like LipidSig [133], LIPID MAPS [134] provide comprehensive, continuously updated computational pipelines and databases tailored specifically for the lipidomics research communities. Moreover, the NCI’s Clinical Proteomic Tumor Analysis Consortium (CPTAC) has integrated mass-spectrometry-based proteomic data with lipidomics and metabolomics data across various cancers, including breast, renal, and colon cancers, thereby enabling detailed correlations between lipid profiles, protein expressions, and genomic alterations [135]. Multi-omics approaches have been instrumental in elucidating the functional roles of lipidation. For example, studies combining differential proteomics and transcriptomics have shown that *N-*myristoylation regulates mitochondrial respiratory complex I, thereby impacting cellular energy metabolism and signaling [136]. Another study integrated genomics, transcriptomics, immunomics, and proteomics, offering strong data support for understanding the role of *S-*palmitoylation in cancer [137]. As multi-omics integration becomes a trend in lipidation research, researchers can study lipidation processes more comprehensively, enhancing our understanding of its role in cellular functions and disease. Moreover, multi-omics data integration has provided a clearer view of the complex interactions between lipidation and other post-translational modifications, such as lipidation with phosphorylation and ubiquitination, which are critical for tumor signaling pathways. For example, studies show that ZDHHC3/9 modulates the tumor immune microenvironment by inhibiting PD- 1/L1 ubiquitination [21, 138], while our lab has found that ZDHHC20 stabilizes FASN by preventing its ubiquitin–proteasome mediated degradation, promoting liver cancer progression [110]. Phosphorylation also plays a role in the *S-*palmitoylation of NLRP3, regulating inflammasome activation and impacting immune responses and tumorigenesis [139].

#### Artificial intelligence

As multi-omics data continues to grow in scale and complexity, managing, integrating, and analyzing this information pose significant challenges. Artificial Intelligence (AI) in medicine plays a critical role in addressing these challenges by enabling multi-layer data integration, predictive modeling, and drug discovery [140]. One such advancement is multiDGD, a deep generative model that processes transcriptomic and epigenomic data, detects statistical associations between genes and regulatory regions, and reveals the relationship between gene expression and epigenetic regulation [141]. AI has proven particularly valuable in lipidation research, allowing for the prediction of modification sites and the discovery of novel inhibitors. For instance, ISM6331, identified through AI-generated models, was found to be a potent TEAD *S-*palmitoylation inhibitor, demonstrating reversible binding to TEAD *S-*palmitoylation sites and strong suppression of TEAD transcriptional activity [142]. Notably, ISM6331 has received orphan drug designation and entered Phase I clinical trials (NCT06566079), with the first patient dosed in January 2025.

Additionally, AI-based tools like SwissPalm2 [143] and deep learning-based graphical models [144] have been developed to predict *S-*palmitoylation sites in proteins, expanding computational capabilities in lipidation studies. AI has also advanced research on *N-*myristoylation and *S-*prenylation. A recent study leveraged machine learning models—including extreme learning machines, incremental feature selection, and maximum relevance minimum redundancy —to develop an optimal *N-*myristoylation site prediction model, enhancing the identification, understanding, and large-scale analysis of *N-*myristoylation in protein sequences [145]. Similarly, a machine learning-based approach to enhance *S-*prenylation site prediction, analyzing both canonical (prenylated, cleaved, and carboxymethylated) and shunted (prenylation-only) motifs, covering 8,000 possible Cxxx sequence combinations [146].

AI is also transforming cancer metabolism research by identifying metabolic enzyme targets that influence both tumor growth and immunity. Using BipotentR, an AI-based multi-omics analysis tool, researchers identified ESRRA (estrogen-related receptor α) as a key regulator of tumor metabolism and immune suppression. Inhibiting ESRRA effectively blocked lipid oxidation in tumor cells, leading to cancer cell death while simultaneously enhancing anti-tumor T cell responses in preclinical models [147]. AI technology is also advancing in drug discovery [148] and bioinformatics, enabling the identification of disease biomarkers [149] and optimizing lipid nanoparticle formulations to improve drug delivery efficiency [150]. These advancements demonstrate that AI holds great potential for lipidation-related disease treatment by enhancing the quantitative analysis, dynamic tracking, and drug development of lipidation.

## Roles of lipidation in the TME

### Tumor cell survival and death signaling

The TME is a complex ecosystem where tumor cells must balance proliferation, metabolic adaptation, and resistance to various forms of cell death to sustain malignancy [151]. Lipidation multiple facets of tumor biology, including signaling, metabolism, and stress adaptation, allowing cancer cells to evade apoptosis, ferroptosis, and other cell death mechanisms [152]. By fine-tuning protein localization, stability, and function, lipidation plays a central role in determining tumor cell fate [4].

#### Tumor cell proliferation and signal transduction

Uncontrolled proliferation is a defining feature of cancer, supported by dysregulated oncogenic signaling and disrupted cell cycle checkpoints [153]. Lipidation modifies key signaling molecules that drive cell division, growth factor receptor activation, and mitotic progression.

**Fig. 4:**
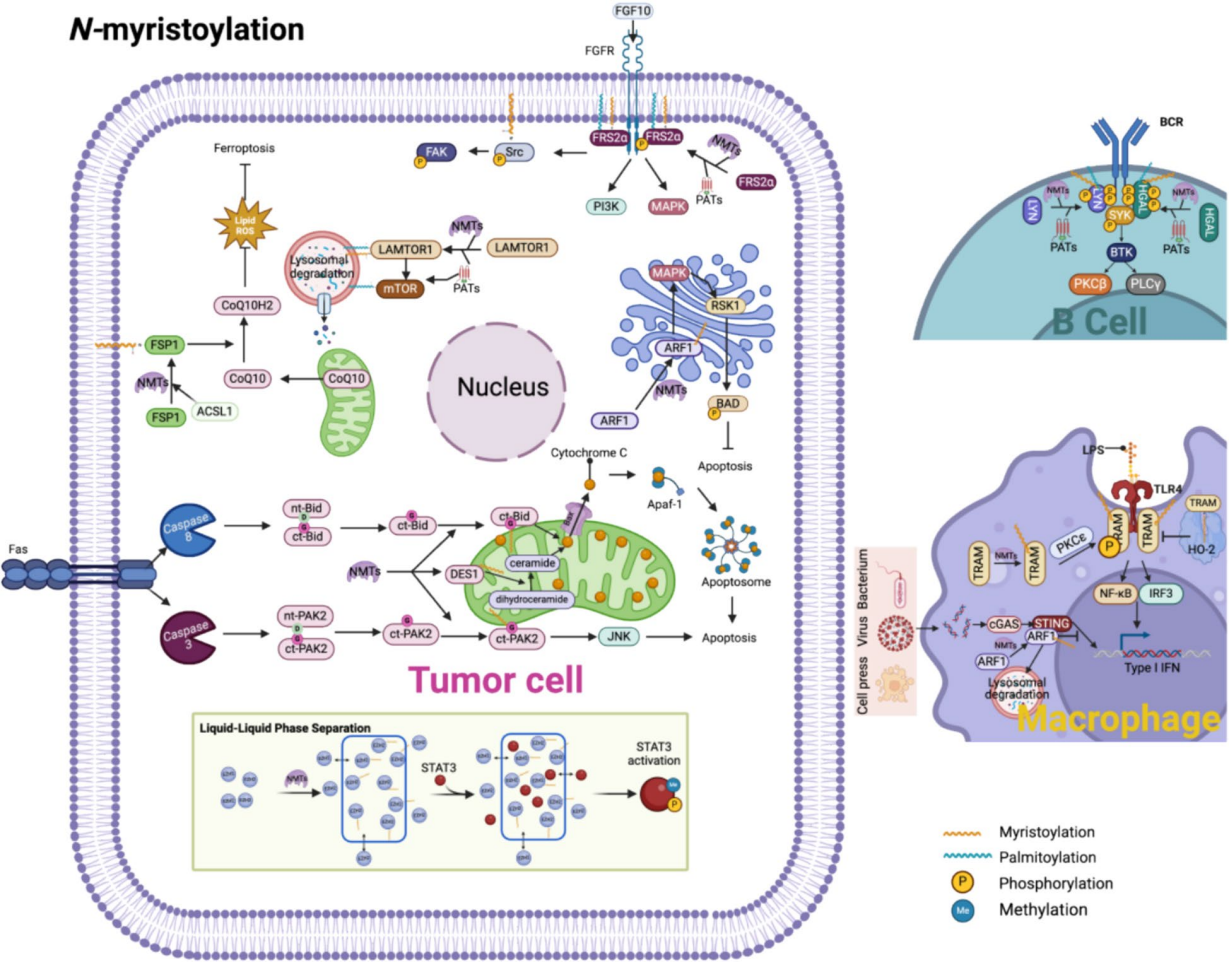
The role of *N-*myristoylation in the TME. This figure depicts comprehensive signaling networks modulated by protein *N-*myristoylation within the tumor microenvironment (TME). Detailed mechanisms include: 1. ARF1-mediated apoptosis suppression: *N-*myristoylation-dependent membrane localization of ADP-ribosylation factor 1 (ARF1) activates ribosomal protein S6 kinase 1 (RSK1), leading to phosphorylation and inhibition of the pro-apoptotic protein Bcl- 2-associated death promoter (BAD). This cascade negatively regulates apoptosis, enhancing tumor cell survival. 2. FGF10/FGFR/Src pathway: Myristoylation of Src kinase facilitates its stable anchoring to lipid raft microdomains, where it promotes fibroblast growth factor receptor (FGFR)-mediated phosphorylation of focal adhesion kinase (FAK). This signaling enhances cell adhesion, migration, and prostate tumor progression driven by paracrine signaling from fibroblast growth factor 10 (FGF10). 3. ARF1/STING-mediated autophagy: Myristoylation-dependent membrane recruitment of ARF1 potentiates stimulator of interferon genes (STING)-mediated autophagy flux, modulating innate immunity and tumor-immune cell interactions. This regulatory axis represents a novel immune evasion strategy exploited by tumors. 4. Apoptosis via PAK2 and Bid: Upon apoptosis induction, *N-*myristoylated caspase-cleaved C-terminal fragments of p21-activated kinase 2 (PAK2)and the pro-apoptotic protein BH3-interacting domain death agonist (Bid) efficiently translocate to mitochondrial membranes. This translocation accelerates cytochrome C release, activating the apoptosome and driving apoptosis through the c-Jun N-terminal kinase (JNK) pathway. 5. Autophagy via LAMTOR1 and mTORC1: *N-*myristoylation and *S-*palmitoylation of late endosomal/lysosomal adaptor, MAPK, and MTOR activator 1 (LAMTOR1) facilitate its lysosomal targeting, essential for the recruitment and activation of mechanistic target of rapamycin complex 1 (mTORC1). This regulatory pathway critically controls autophagy initiation, influencing cellular metabolism and survival within the nutrient-stressed TME. 6. Ferroptosis regulation via ACSL1-FSP1: Acyl-CoA synthetase long-chain family member 1 (ACSL1)-dependent *N-*myristoylation of ferroptosis suppressor protein 1 (FSP1) targets it to the plasma membrane, enhancing coenzyme Q10 (CoQ₁₀) reduction activity. This mechanism reduces lipid peroxide accumulation and lipophilic radical formation, protecting cancer cells from ferroptotic cell death. 7. Toll-like receptor 4 (TLR4) signaling activation via TRAM: Following stimulation with lipopolysaccharide (LPS) or pathogen-associated molecular patterns (PAMPs), myristoylated TRIF-related adaptor molecule (TRAM) rapidly relocates to plasma membrane microdomains. Protein kinase C epsilon (PKCε) phosphorylates TRAM, enhancing its binding affinity for the adaptor protein TIR-domain-containing adapter-inducing interferon-β (Trif). This complex subsequently activates interferon regulatory factor 3 (IRF3)-mediated type I interferon (IFN-I) production and nuclear factor-κB (NF-κB)-dependent proinflammatory cytokine secretion, orchestrating macrophage-driven inflammation within the TME. 8. B-cell receptor (BCR) signaling enhancement via Lyn kinase: Myristoylation and subsequent -palmitoylation of the Src-family kinase Lck/Yes-related novel protein tyrosine kinase (Lyn) stabilize B-cell receptor (BCR) complexes within lipid raft domains on the plasma membrane. This dual lipidation enhances BCR clustering, antigen recognition, and downstream signaling cascades mediated by phospholipase C gamma (PLCγ), augmenting adaptive immune responses within the TME. 9. ARF1-STING axis (extended detail): *N-*myristoylation of ARF1 enhances membrane association and subsequent interaction with STING, promoting STING aggregation and downstream activation of autophagy, shaping the tumor-immune interface. 10. Liquid–liquid phase separation (LLPS) via EZH2 and STAT3: Enhancer of zeste homolog 2 (EZH2)-mediated liquid–liquid phase separation (LLPS) is enhanced by *N-*myristoylation-driven hydrophobic interactions, facilitating formation of membraneless condensates. These condensates efficiently concentrate activators of signal transducer and activator of transcription 3 (STAT3), promoting STAT3 phosphorylation and subsequent transcriptional activation of oncogenic genes critical for tumor proliferation, immune evasion, and epigenetic reprogramming. ACSL1: Acyl-CoA synthetase long-chain family member 1; ARF1: ADP-ribosylation factor 1; BAD: Bcl- 2-associated death promoter; BCR: B-cell receptor; BTK: Bruton’s tyrosine kinase; CoQ₁₀: Coenzyme Q10; ct-Bid: Cleaved C-terminal fragment of BH3-interacting domain death agonist; ct-PAK2: Cleaved C-terminal fragment of p21-activated kinase 2; EZH2: Enhancer of zeste homolog 2; FAK: Focal adhesion kinase; Fas: Fas cell surface death receptor; FGF10: Fibroblast growth factor 10; FGFR: Fibroblast growth factor receptor; FRS2α: Fibroblast growth factor receptor substrate 2-alpha; FSP1: Ferroptosis suppressor protein 1; IFN: Interferon; IRF3: Interferon regulatory factor 3; JNK: c-Jun N-terminal kinase; LAMTOR1: Late endosomal/lysosomal adaptor, MAPK and MTOR activator 1; LPS: Lipopolysaccharide; Lyn: Lck/Yes-related novel protein tyrosine kinase; mTOR: Mechanistic target of rapamycin; NFKB: Nuclear factor-κB; NMT: *N-*myristoyltransferase; nt-Bid: N-terminal fragment of BH3-interacting domain death agonist; nt-PAK2: N-terminal fragment of p21-activated kinase 2; PAT: Protein acyltransferase; PAK2: p21-activated kinase 2; PKC-β: Protein kinase C beta; PKCε: Protein kinase C epsilon; PLCγ: Phospholipase C gamma; STAT3: Signal transducer and activator of transcription 3; STING: Stimulator of interferon genes; TLR4: Toll-like receptor 4; TRAM: TRIF-related adaptor molecule

NMT1, which is upregulated in various cancers, including oral squamous cell carcinoma (OSCC) [154], liver cancer [155], lung cancer [19], breast cancer (BrCa) [156], osteosarcoma [157], and bladder cancer [158], is frequently associated with poor prognosis [159]. As one of the earliest identified myristoylated proteins, Src is crucial in mediating ECM-integrin interactions, linking the ECM to the cytoskeleton, and inducing cell adhesion turnover [160]. In the TME, Src *N-*myristoylation, particularly when enhanced by exogenous myristic acid, promotes the activation of the FAK-Src signaling axis [161] (Fig. 4). This leads to an amplification of FAK phosphorylation and yes-associated protein (YAP)/transcriptional coactivator with PDZ-binding motif (TAZ)-dependent IL- 6 expression, reinforcing the tumor-stroma crosstalk. Src *N-*myristoylation also facilitates the secretion of Periostin by fibroblasts, contributing to ECM remodeling and sustaining the TME homeostasis through an IL- 6/STAT3 feedback loop, which ultimately promotes colorectal cancer progression [162]. The mitogen-activated protein kinase (MAPK)/ERK pathway, a key regulator of tumor proliferation, is initiated when receptor tyrosine kinases (RTKs), such as epidermal growth factor receptor (EGFR) or fibroblast growth factor receptor (FGFR), activate RAS GTPases. Upon activation, RAS localize to the PM to engage RAF kinases, triggering ERK phosphorylation and subsequent transcription of cell cycle-promoting genes [163]. The membrane anchoring of RAS is controlled by *S-*prenylation and *S-*palmitoylation (Fig. 6). Farnesylation at the CAAX motif facilitates the initial recruitment of RAS to the PM, but stable retention of HRAS and NRAS requires *S-*palmitoylation [86, 87]. They translocate to the membrane and initiate the Ras-Raf-MEK-ERK pathway [164, 165]. The absence of *S-*prenylation in certain RAS-related inhibitors prevents their membrane localization, thereby impairing their pro-tumorigenic effects [166].

Lipidation also regulates the phosphoinositide 3-kinase (PI3 K)/AKT/mTOR pathway, which controls cell growth, survival, and metabolic adaptation [167]. KRAS-dependent activation of PI3 K relies on its *S-*palmitoylation, which enhances its interaction with PI3 K and promotes AKT activation. ZDHHC13 [168] and ZDHHC20 [169, 171] mediated *S-*palmitoylation sustain PI3 K/AKT signaling through EGFR, driving uncontrolled proliferation. ABHD17 C affects the PI3 K/AKT signaling pathway by causeing cell cycle arrest, and reducing cell proliferation [172]. Downstream of AKT, mTORC1 activation is controlled by Rheb, whose *S-*prenylation is required for its lysosomal localization and function [172, 173]. When PDEδ, a *S-*prenylation chaperone, is inhibited, Rheb mislocalizes, leading to defective mTORC1 activation and impaired tumor growth [174]. Disruption of upstream and downstream events in *S-*prenylation, such as inhibition of the mevalonate (MVA) pathway or PDEδ, leads to the arrest of growth and induced cell death [164, 165]. Additionally, *S-*palmitoylation differentially affects mTOR signaling depending on cancer type: in renal cell carcinoma, ZDHHC2 enhances AGK *S-*palmitoylation, leading to AKT-mTORC1 activation [175], whereas in BrCa, ZDHHC22 reduces mTOR stability, thereby suppressing AKT-mTOR signaling [176].

The Hippo pathway is a highly conserved regulatory network regulates cell proliferation by YAP/TAZ-TEA domain family member (TEAD) transcriptional activity [177]. TEAD auto-palmitoylation is essential for its stability and interaction with YAP/TAZ, driving proliferative gene expression [178, 179]. Given its role in sustaining uncontrolled growth, inhibiting TEAD *S-*palmitoylation has emerged as a promising strategy for blocking Hippo pathway activation in cancers.

Additionally, *N-*myristoylation promotes or stabilizes liquid–liquid phase separation (LLPS), imparting hydrophobicity to proteins and increasing their signal transduction efficiency [180, 181]. In lung cancer, the *N-*myristoylation of enhancer of zeste homolog 2 (EZH2) facilitates the formation of LLPS droplets that concentrate the substrate signal transducer and transcription 3 (STAT3) activator, thereby increasing STAT3 signaling and driving cell proliferation [180](Fig. 4).

Interestingly, the effects of lipidation on tumor progression vary depending on the tissue type, developmental stage, and signaling environment. For instance, *S-*prenylation-related enzyme deletion can have paradoxical outcomes: inactivation of Rce1 can promote myeloproliferative diseases [182], whereas conditional ablation of ICMT may impair oncogenicity mediated by all RAS isoforms [183]. In some instances, concurrent deactivation of KRAS G12D and ICMT may accelerate disease progression in embryonic pancreatic cancer models [184]. Different conclusions hinge on the tissue type, stage of development, and signaling environment.

Lipidation also plays a role in cell cycle regulation and mitotic checkpoint control. Cell cycle regulation determines whether cells continue to proliferate or enter cell death pathways. NMT inhibitors induce cell cycle arrest by blocking the PI3 K and MAPK signaling pathways [185, 186], whereas the myristylation of sphingosine kinase 1 delays exit from the G₀/G₁ phase [17]. Additionally, centromere proteins CENP-E and CENP-F, expressed during mitosis to mediate the G2 to M checkpoint, are regulated by *S-*prenylation, which modulates their interaction with the RZZ complex and ensures proper chromosome segregation while inhibiting farnesylation of the dynein adaptor Spindly causes mitotic defects and results in metaphase arrest, preventing chromosome separation [187–189]. Blocking *S-*prenylation upregulates the expression of the cyclin-dependent kinase (CDK) inhibitors p21 and p27, inhibits the activity of CDK2 and the expression of cyclins D1 and E2, and blocks the MAPK/ERK and PI3 K/Akt pathways, thus halting cell cycle progression [190–195]. This inhibition also increases DNA damage and induces cellular senescence [196].

#### Tumor metabolism and energy adaptation

Cancer cells undergo metabolic reprogramming to meet their high demands for adenosine triphosphate (ATP), lipids, and metabolic intermediates, enabling them to adapt to metabolic stress [197]. Some mitochondrial proteins require *N-*myristoylation for proper mitochondrial targeting and respiratory chain assembly [198, 199], ensuring efficient oxidative phosphorylation (OXPHOS) and ATP production [136]. For instance, NDUFB7, an accessory subunit of mitochondrial complex I, and DMAC1, a critical assembly factor, require *N-*myristoylation for their mitochondrial targeting and function [15]. *N-*myristoylation also regulates the mitochondrial localization of proteins like Cyb5R3 and DES1, influencing sphingolipid metabolism and altering cancer cell metabolism [200–202]. Similarly, *N-*myristoylation at the N-terminus of Mic19 enhances its interaction with the mitochondrial outer membrane and Tom20, facilitating its efficient import into the mitochondrial intermembrane space [203]. Inhibition of NMT1 leads to mitochondrial dysfunction and energy crisis, ultimately leading to cancer cell death. Further, the *N-*myristoylation of AMPK enables metabolic plasticity, supporting the transition from OXPHOS to glycolysis. Inhibition of AMPK *N-*myristoylation prevents the metabolic adaptation of cancer cells, making them more susceptible to cell death [204, 205].

**Fig. 5:**
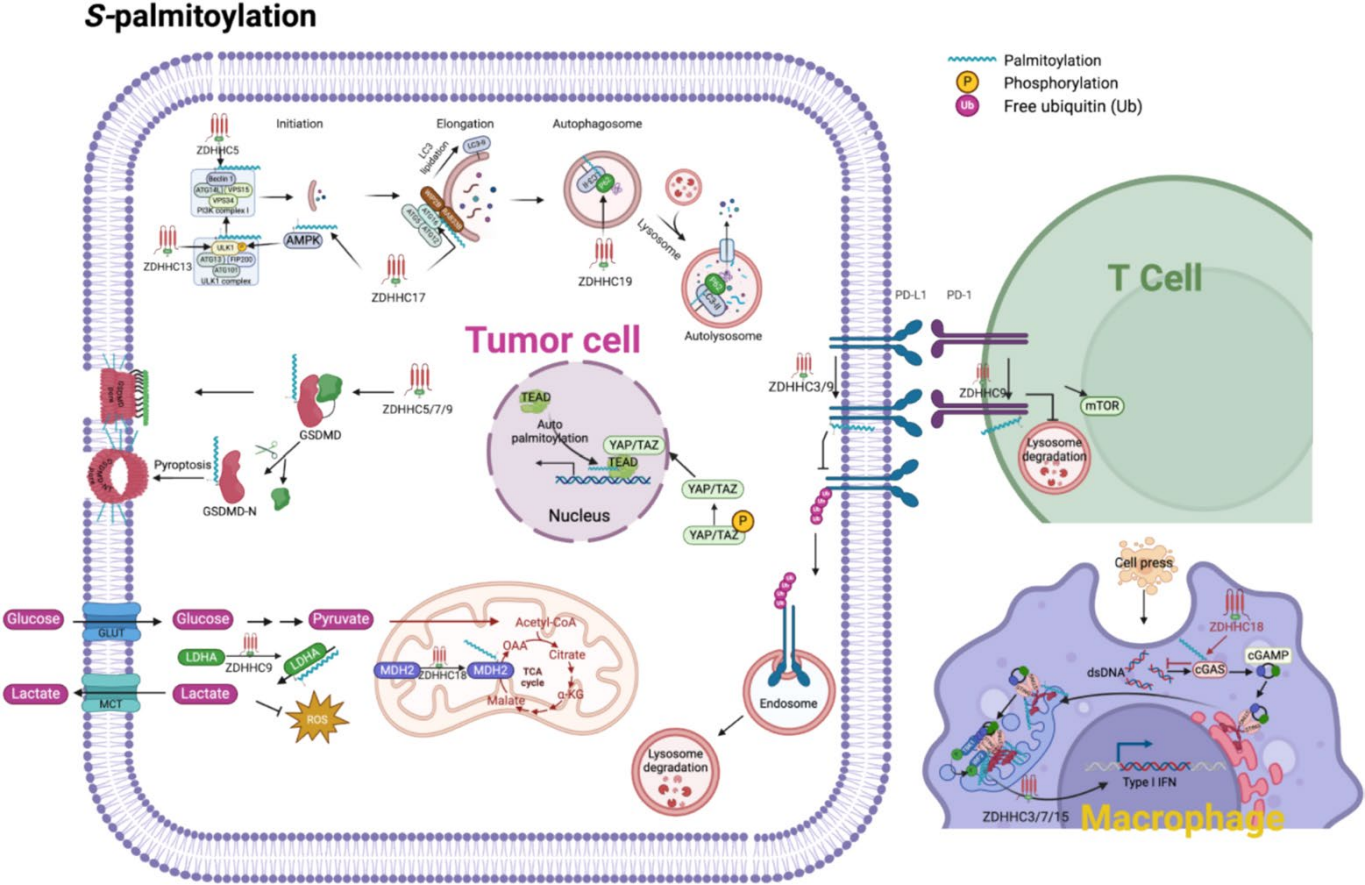
The role of *S-*palmitoylation within the TME. *S-*palmitoylation influences several critical cellular processes across different cell types in the TME. 1.Hippo Pathway: Upon dephosphorylation, Yes-associated protein (YAP) and transcriptional coactivator with PDZ-binding motif (TAZ) translocate into the nucleus, where they selectively interact with *S-*palmitoylated TEA domain transcription factor (TEAD) proteins. This YAP/TAZ-TEAD transcriptional complex activates oncogenic gene expression, promoting tumor cell proliferation, survival, and therapeutic resistance. 2. Autophagy: *S-*palmitoylation controls multiple steps of the autophagy cascade. Under nutrient deprivation or mechanistic target of rapamycin (mTOR) inhibition, AMP-activated protein kinase (AMPK) phosphorylates unc- 51-like kinase 1 (ULK1) at Ser317 and Ser777, enhancing ULK1 activation. Simultaneously, Zinc finger DHHC-type containing 13 (ZDHHC13)-mediated *S-*palmitoylation facilitates ULK1 recruitment to autophagosome formation sites, amplifying its ability to phosphorylate autophagy-related 14-like protein (ATG14L) in the class III phosphatidylinositol 3-kinase (PI3 K) complex. Additionally, ZDHHC5-mediated Beclin 1S-palmitoylation enhances its interaction with ATG14L and vacuolar protein sorting 15 (VPS15), synergistically activating PI3 K lipid kinase activity to initiate autophagosome formation. ZDHHC17 primes AMPK activation, forming a feedforward loop essential for sustained autophagic flux. During autophagosome elongation, ZDHHC17-mediated ATG16L1S-palmitoylation stabilizes its interactions with WD repeat domain phosphoinositide-interacting protein 2B (WIPI2B) and Rab33B, ensuring efficient LC3 lipidation (LC3-II) and autophagosome maturation. Finally, the selective autophagy receptor p62/SQSTM1 is palmitoylated by ZDHHC19, enhancing its LC3-II affinity, thereby facilitating the degradation of ubiquitinated cargo via lysosomal fusion. 3. Pyroptosis: *S-*palmitoylation of gasdermin D (GSDMD) at cysteine 191 (C191), catalyzed by ZDHHC5, ZDHHC7, and ZDHHC9, facilitates its membrane localization and pore formation in response to NLR family pyrin domain containing 3 (NLRP3) inflammasome activation. While gasdermin D N-terminal domain (GSDMD-N) was previously thought to require cleavage for activation, new evidence suggests that *S-*palmitoylation alone can prime uncleaved GSDMD for membrane pore formation, establishing a novel checkpoint for pyroptosis regulation. 4. Energy Metabolism Regulation: ZDHHC9-mediated *S-*palmitoylation of lactate dehydrogenase A (LDHA) enhances its catalytic activity, driving aerobic glycolysis (Warburg effect), increasing lactate secretion, and reducing reactive oxygen species (ROS) accumulation by shifting metabolism away from mitochondrial oxidative phosphorylation (OXPHOS). This metabolic adaptation supports tumor growth, invasion, and acidifies the TME. Additionally, *S-*palmitoylation of malate dehydrogenase 2 (MDH2) at C138 enhances its enzymatic activity, boosting ATP production and facilitating tumor cell proliferation under metabolic stress conditions. 5. Immune Checkpoint: *S-*palmitoylation of programmed death-ligand 1 (PD-L1) prevents its monoubiquitination, protecting it from lysosomal degradation via the endosomal sorting complex required for transport (ESCRT) pathway. This stabilization prolongs PD-L1 surface expression, enabling persistent T-cell suppression by engaging programmed death- 1 (PD- 1) on tumor-infiltrating lymphocytes (TILs). Similarly, *S-*palmitoylation of PD- 1 prevents its degradation, stabilizing PD- 1 inhibitory signaling, which enhances mechanistic target of rapamycin (mTOR) activation, ultimately promoting tumor survival, proliferation, and immune evasion. 6. cGAS-STING Pathway: *S-*palmitoylation of cyclic GMP-AMP synthase (cGAS) inhibits DNA binding, leading to reduced 2′3'-cyclic GMP-AMP (cGAMP) synthesis and weakening stimulator of interferon genes (STING) activation, thereby suppressing innate immune responses. However, *S-*palmitoylation of STING promotes its Golgi aggregation, a prerequisite for TANK-binding kinase 1 (TBK1) activation and interferon regulatory factor 3 (IRF3) phosphorylation, ultimately enhancing the type I interferon (IFN-I) response. AMPK: AMP-activated protein kinase; ATG14L: Autophagy-related 14-like protein; ATG16L1: Autophagy-related protein 16-like 1; Beclin 1: Bcl- 2-interacting coiled-coil protein; cGAMP: 2′3'-cyclic GMP-AMP; cGAS: Cyclic GMP-AMP synthase; GSDMD: Gasdermin D; LDHA: Lactate dehydrogenase A; LC3-II: Microtubule-associated protein 1 light chain 3-II; mTOR: Mechanistic target of rapamycin; MDH2: Malate dehydrogenase 2; p62/SQSTM1: Sequestosome- 1; PD- 1: Programmed death- 1; PD-L1: Programmed death-ligand 1; ROS: Reactive oxygen species; STING: Stimulator of interferon genes; TEAD: TEA domain transcription factor; ULK1: Unc- 51-like kinase 1; VPS15: Vacuolar protein sorting 15; WIPI2B: WD repeat domain phosphoinositide-interacting protein 2B; YAP: Yes-associated protein; ZDHHC: Zinc finger DHHC-type containing protein

*S-*palmitoylation is crucial in regulating tumor cell glycolysis and metabolic adaptation, particularly its impact on lactate dehydrogenase A (LDHA) [16] (Fig. 5). LDHA catalyzes the conversion of pyruvate to lactate, a critical step in glycolysis, especially under hypoxic conditions [206]. LDHA levels are elevated in tumors, mainly due to their role in driving lactate production while bypassing mitochondrial OXPHOS. This metabolic shift enables cancer cells to thrive in hypoxic environments, promoting tumor growth and invasion [207]. Increased lactate output further acidifies the TME, supporting tumor invasion and metastasis [208]. Additionally, *S-*palmitoylation indirectly affects the production of ROS by modulating glycolysis. The shift from mitochondrial OXPHOS to glycolysis reduces ROS generation, producing less ROS than mitochondrial respiration [209]. Thus, the *S-*palmitoylation of LDHA for tumor cells minimizes oxidative stress, allowing cell survival and proliferation, especially under metabolic or hypoxic stress conditions [206]. MDH2 is a key enzyme in the TCA cycle, activated by *S-*palmitoylation at position C138. This modification enhances the catalytic activity of MDH2, promotes ATP production, and supports tumor cell proliferation [210]. Therefore, *S-*palmitoylation is a "metabolic switch" that helps cancer cells survive and proliferate in hypoxic or nutrient-poor environments. Therefore, inhibition of the specific PATs of MDH2 or LDHA may be a promising therapeutic strategy to target tumors. However, studies have shown that depalmitoylated KRAS4 A promotes glycolysis through its interaction with hexokinase 1, thereby meeting the increased energy demands required for tumor proliferation. This process is closely correlated with the copy number of mutant KRAS, independent of the MAPK signaling pathway [211]. Regarding lipid metabolism, *S-*palmitoylation regulates endothelial lipase and the peroxisome proliferator-activated receptor gamma–ATP citrate lyase axis through *S-*palmitoylation [212, 213]. Notably, the downregulation of acyl-CoA oxidase 1 modulates the accumulation of palmitic acid (PA), subsequently affecting the *S-*palmitoylation of β-catenin and influencing tumor progression [214].

The *S-*prenylation-related process affects tumor metabolism. TCS Blockade of the MVA pathway leads to reduced levels of coenzyme Q10, a critical electron transport protein in the mitochondrial respiratory chain, and polyisoprenoids, vital free radical scavengers in the cell membrane. This disruption causes a decrease in mitochondrial membrane potential, promotes the release of pro-apoptotic factors, and induces mitochondrial dysfunction, thus exacerbating oxidative stress And inhibiting Rab protein *S-*prenylation can induce the unfolded protein response and disrupt purine metabolism [215]. Inhibition of ICMT severely affects mitochondrial function and inhibits ATP production by affecting electron transport chains I, II, and III. Inadequate energy supply leads to a decrease in key metabolites in the TCA, inhibiting anabolic processes and cell growth. Particularly in ICMT-inhibitor-sensitive pancreatic cancer cells, ICMT inhibition causes mitochondrial respiratory defects and energy depletion, leading to a significant upregulation of p21. p21 serves as a sensor of cellular energy depletion and activates BNIP3 to trigger the apoptotic response [216, 217].

#### Tumor cell stress responses and cell death regulation

##### Autophagy

Autophagy is a vital cellular process that maintains homeostasis in cancer cells through lysosomal degradation mechanisms. It plays a dual role in cancer, acting both as a tumor suppressor by clearing damaged organelles and as a tumor promoter by sustaining metabolic adaptation and survival under stress [218].

Autophagy is tightly linked to mechanistic target of rapamycin complex 1 (mTORC1), a central regulator of nutrient sensing and cellular metabolism [219]. *N-*myristoylation and *S-*palmitoylation fine-tune the balance between mTORC1 activity and autophagy initiation. *N-*myristoylation and *S-*palmitoylation stabilizes lysosomal recruitment of LAMTOR1, a key component of the Ragulator complex, which activates mTORC1 under nutrient-rich conditions [158, 220](Fig. 4). Inhibiting *N-*myristoylation of LAMTOR1 disrupts mTORC1 localization, impairing its ability to suppress autophagy [83]. AMPK *N-*myristoylation enhances mitophagy [221, 222], while ARF1 *N-*myristoylation activates stimulator of interferon (IFN) gene (STING)-dependent autophagy [223], contributing to metabolic adaptation in cancer cells. Conversely, *N-*myristoylation of protein kinase B (AKT) suppresses autophagy by reducing Beclin- 1, LC3a, and LC3b expression while increasing p62 levels, thereby shifting the cell towards survival instead of degradation [224]. In BrCa, blocking NMT1 increases protein degradation and ER stress, triggers the c-Jun N-terminal kinase (JNK) pathway, and stimulates autophagy, thus contributing to tumorigenesis [156].

Autophagy initiation requires the unc- 51-like kinase (ULK) and class III PI3 K complexes [225], with ULK1 acting as a central regulatory hub. Upon nutrient deprivation or mammalian target of rapamycin (mTOR) inhibition, ULK1 activation is triggered through dual regulatory mechanisms: AMPK-mediated phosphorylation (Ser317/Ser777) directly enhances its kinase activity [226], while ZDHHC13-dependent *S*-palmitoylation facilitates ULK1 complex translocation to the autophagosome formation site [227] (Fig. 5). This modification not only complements AMPK-driven phosphorylation but also potentiates ULK1's ability to phosphorylate ATG14L within the VPS34 complex, thereby amplifying PI3 K lipid kinase activity – a mechanism evolutionarily conserved from C. elegans to mammals [227, 228]. Intriguingly, ZDHHC17 further modulates this network by priming AMPK activation [229]. Creating a feedforward loop that ensures robust autophagy initiation. Recent studies challenge the linear view of AMPK as a simple "on-switch", revealing its context-dependent roles: while sustaining basal autophagy, AMPK paradoxically attenuates acute autophagic responses under prolonged energy stress [230]. Moreover, DHHC5-mediated *S-*palmitoylation of Beclin 1 enhances its interaction with ATG14L and VPS15, further promoting autophagy [231]. After the initial stages of autophagy, *S-*palmitoylation promotes autophagosome formation by modifying ATG16L1 [232–234]. This modification promotes the interaction of ATG16L1 with WIPI2B and Rab33B, thereby ensuring efficient autophagosome generation [233]. Even calcium homeostasis during autophagy is regulated via *S-*palmitoylation of MCOLN3/TRPML3, a Ca^2^⁺-permeable cation channel [235]. On the other hand, PPT1 deficiency disrupts the removal of *S-*palmitoylation, affecting inositol 1,4,5-trisphosphate receptor 1 expression and thereby dysregulating lysosomal Ca homeostasis [236]. Moreover, PPT1 deficiency disrupts Rab7 localization and impairs autolysosome function [237]. The selective autophagy receptor p62 and autophagy substrates are also regulated by *S-*palmitoylation, further refining the selectivity and efficiency of the autophagic process. Specifically, p62 is palmitoylated by ZDHHC19 to increase its affinity for LC3-positive autophagosomes, thus strengthening the selective degradation of ubiquitinated proteins [238]. In contrast, ZDHHC5 prevents NOD2 P62-mediated degradation by maintaining its membrane localization [239].

An *S-*prenylation-related growth-promoting protein PRL- 3 drives the PIK3 C3-Beclin- 1-dependent autophagosome formation to accelerate cell autophagy [240]. Autophagy is negatively regulated by the ICMT [241]. ICMT inhibition induces autophagy and acts as an upstream event of apoptosis in some tumors. This suggests that targeting ICMT can activate a cascade of cellular events that promote cell death. The synergistic effect of both processes can be achieved via ICMT inhibitor cysmethynil, resulting in substantial antitumor effects [242]. ICMT inhibition also suppresses mitochondrial respiration, leading to cellular energy depletion and increased autophagy, thus providing further insight into how ICMT inhibition contributes to autophagy regulation [216].

##### Apoptosis

Apoptosis is a programmed cell death mechanism that eliminates damaged or stressed cells without triggering inflammation. This process is tightly regulated by caspase activation, cytochrome C release, and pro-apoptotic signaling cascades [243]. Apoptosis is triggered through caspase-mediated proteolytic cleavage, predominantly via glycine residues, to eliminate damaged cells with the advantage of not inducing an inflammatory response [244]. Posttranslational *N-*myristoylation occurs when caspases cleave proteins, exposing an internal glycine residue that serves as a lipidation site (Fig. 4). Modifying the resulting C-terminal fragment is crucial for balancing cell survival and apoptotic pathways [245–247]. *N-*myristoylation plays a dual role in apoptosis, influencing both pro-apoptotic and anti-apoptotic pathways. *N-*myristoylation of proapoptotic proteins, including C-terminal BID (ctBID) [248] and C-terminal p21-activated protein kinase 2 (ctPKA2) [249], targets them to mitochondrial membranes and cristae, increasing cytochrome C release and activating the JNK pathway, thereby promoting apoptosis. Conversely, the *N-*myristoylation of antiapoptotic proteins, such as ctGelsolin [250] and ctPKC [251], inhibits apoptosis [252]. *N-*myristoylation also regulates apoptosis through apoptosis-related proteins. For example, ARF1 *N-*myristoylation negatively regulates apoptosis by activating RSK1 and phosphorylating BAD [253, 254]. *N-*myristoylation of FUS1 induces apoptosis by activating Bcl- 2-associated X (BAX)and caspases [255]. In addition, the *N-*myristoylation of DES1 targets it to the mitochondria, affecting mitochondrial sphingolipid metabolism, particularly ceramide production. Ceramide facilitates cytochrome C release and disrupts the electron transport chain [200, 256, 257]. Interestingly, caspases regulate the cleavage and relocalization of NMTs. In cancer cells with diminished caspase activity, NMT cleavage is impaired, leading to persistently high NMT expression, which supports anti-apoptotic signaling [258]. Thus, NMT inhibition reduces Bcl- 2 expression and increases cleaved caspase- 3 levels, inducing apoptosis in tumor cells [158, 259].

*S-*palmitoylation modulates apoptotic signaling and thus determines cell fate under conditions of stress [260]. In the extrinsic apoptosis, it enhances apoptotic signal transduction by regulating the membrane localization and oligomerization of TNF superfamily members, such as Fas and DR4 [39, 261, 262]. DR4 in lipid rafts increases the sensitivity of rectal cancer cells to TRAIL-mediated apoptosis [263, 264]. Similarly, *S-*palmitoylation increases Fas-mediated caspase- 8 activation and promotes apoptosis [262, 265]. In endogenous apoptosis, BAX *S-*palmitoylation is necessary for binding to the mitochondrial outer membrane, whereas nonpalmitoylated BAX reduces its apoptotic activity [266]. Moreover, ZDHHC9 palmitoylates immunoglobulin protein, which enhances cell tolerance to ER stress, consequently reducing apoptosis [267]. Consequently, the knockout of ZDHHC9 induces apoptosis in lung and bladder cancer tumor cells [267, 268]. In BrCa, *S-*palmitoylation of neurotensin receptor- 1 enhances its interaction with Gαq/11, thus inhibiting apoptosis [269].

Blocking the MVA pathway inhibits *S-*prenylation, reducing the availability of FPP and GGPP, thereby preventing protein *S-*prenylation and altering apoptotic sensitivity [270, 271]. *S-*prenylation of Rab family proteins (e.g., Rab7) regulates vesicle trafficking. In drug-resistant cancer cells, disrupting Rab7 *S-*prenylation leads to excessive ROS accumulation, which ultimately triggers apoptosis [215, 272]. MVA pathway inhibition simultaneously activates intrinsic apoptotic pathways by activating Bcl- 2, Caspase- 3, PARP, and c-Jun, leading to cancer cell apoptosis [273, 274]. In KRAS*-*mutant cells, *S-*prenylation inhibition also results in severe ER stress [275]. Deprenylation triggers the extrinsic apoptosis pathway by increasing DR5 expression, enhancing TRAIL-mediated apoptotic signaling, activating caspase- 3, and promoting tumor cell death [276].

##### Ferroptosis

Ferroptosis is a nonapoptotic form of cell death characterized by iron-dependent lipid peroxidation [277]. This process involves iron accumulation, lipid peroxidation, ROS production, and depletion of glutathione peroxidase 4 (GPX4) [278]. Owing to their increased iron requirements, cancer cells are particularly vulnerable to ferroptosis, making its regulation a critical determinant of tumor survival [279, 280].

*N-*myristoylation critically regulates ferroptosis by targeting ferroptosis suppressor protein 1 (FSP1) to the PM, increasing the reduction in coenzyme Q₁₀(CoQ10) and reducing the accumulation of lipophilic radicals and lipid peroxides [281]. Mutations at *N-*myristoylation sites on FSP1 impair membrane localization and ferroptosis inhibitory function [282](Fig. 4). In addition, ACSL1-mediated FSP1 *N-*myristoylation increases resistance to ferroptosis in ovarian cancer [283]. *N-*myristoylation also enhances the interaction between icFSP (an FSP1 inhibitor) and FSP1, promoting FSP1 phase separation and impairing tumor growth [284]. *N-*myristoylation inhibition triggers extensive lipid peroxidation and ferroptosis and activates the parthanatos pathway, increasing tumor cell sensitivity to ferroptosis-induced necrotic apoptosis [18, 19].

SLC7 A11 has a crucial role in maintaining cellular antioxidant defense and redox homeostasis by importing cystine for SLC7 A11 glutathione synthesis and regulates ferroptosis by preventing oxidative damage and lipid peroxidation [285]. ZDHHC8 stabilizes SLC7 A11, an essential regulator of ferroptosis, by decreasing its ubiquitination and degradation. This can be further enhanced by the long-chain noncoding RNAs DUXAP8 and AMPK, which inhibit ferroptosis [286, 287]. Moreover, PPT1 upregulates the expression of the antioxidant enzyme GPX4, further inhibiting ferroptosis in OSCC [288] (Fig. 5).

*S-*prenylation regulates ferroptosis primarily through the MVA pathway by modulating the maturation of selenocysteine tRNA, thus affecting GPX4 synthesis and FSP1 activity [289, 290] and influencing ferroptosis occurrence. Products of the MVA pathway, such as isopentenyl pyrophosphate and CoQ10, inhibit lipid peroxidation; therefore, their depletion is believed to induce ferroptosis [289–291].

##### Pyroptosis and inflammasome activation

Pyroptosis is an inflammation-driven, caspase- 1-dependent, and gasdermin-mediated form of programmed cell death. It is characterized by cell swelling and the formation of bubble-like protrusions on the plasma membrane before rupture, leading to the release of inflammatory factors. This process is mediated by inflammasome assembly, which triggers the immune response [292].

Pyroptosis depends on gasdermin D (GSDMD) family proteins, and ZDHHC5/7/9-mediated *S-*palmitoylation of GSDMD at the C191 site facilitates its activation and translocation to the cell membrane after NLRP3 inflammasome cleavage, where it then forms pores and triggers pyroptosis [293]. Whereas prior studies theorized that GSDMD must undergo cleavage to produce the GSDMD N-terminal domain (GSDMD-NT) and create large transmembrane pores, a more recent study challenged this notion by showing that reversible *S-*palmitoylation is an essential checkpoint for pore formation by both GSDMD-NT and intact GSDMD, [294] (Fig. 5). The multiple roles of *S-*palmitoylation show that the depalmitoylating enzyme APT2 promotes the activation of GSDMD by removing palmitoyl groups. Nevertheless, the knockout of ABHD17 C enhances pyroptosis responses in liver cancer cells [295], highlighting the crucial balance between *S-*palmitoylation and depalmitoylation in pyroptosis [293]. Moreover, a *S-*palmitoylation-related GSDMD inhibitor, NU6300, was invented and has an elite effect [296].

The NLRP3 inflammasome comprises NLRP3, apoptosis-associated speck-like protein (ASC), and pro–caspase–1, which collectively facilitate the cleavage and activation of GSDMD [297] (Fig. 4). *S-*palmitoylation represents a crucial regulatory switch in NLRP3 inflammasome activity. Indeed, ZDHHC1 enhances the interaction between NLRP3 and NEK7, promoting inflammasome assembly [298]. ZDHHC5 of NLRP3 increases its degree of membrane binding and stability, further supporting practical inflammasome function. In contrast, ABHD17 A and ZDHHC12 act to prevent hyperactivated inflammatory responses by depalmitoylating or inhibiting NLRP3, thereby preventing overactivation of the inflammasome [299, 300]. Similarly, inhibiting fatty acid synthase (FASN) blocks the *S-*palmitoylation of NLRP3, resulting in reduced inflammasome activation [301].

The geranylgeranylation of RhoA is essential for proper NLRP3 inflammasome assembly [302] ensuring effective GSDMD-dependent pyroptotic signaling [303]. Additionally, reduced *S-*prenylation of Rac1 enhances its interaction with IQGAP1, further stimulating NLRP3 inflammasome activation and promoting pyroptosis [304].

### Cell plasticity and dynamic behavior

Epithelial-mesenchymal transition (EMT) and MET are no longer viewed as binary processes but as dynamic transitions where cells can adopt hybrid states with both epithelial and mesenchymal traits, exhibiting epithelial-mesenchymal plasticity [305]. This plasticity allows tumor cells to adapt and respond to various microenvironmental cues, increasing their capacity for invasion and metastasis.

For instance, N-myristoylation plays a crucial role in EMT by modulating the FGF/FGFR signaling axis. The transition between FGFR2 IIIb and FGFR2 IIIc isoforms, which is key to mesenchymal transformation, is influenced by the AKT3/IWS1 pathway [306, 307]. The scaffold protein FRS2α, a key mediator of FGF signaling, undergoes *N-*myristoylation, which stabilizes FGFR/Src interactions, amplifying downstream signaling and promoting EMT plasticity [186](Fig. 4). Concurrently, *S-*palmitoylation of transcriptional regulators such as Snail reprograms lipidation networks within EMT by activating APT2 [308], a depalmitoylating enzyme that displaces Scribble from the PM, disrupting Hippo-YAP signaling and enhancing the MAPK pathway—key drivers of mesenchymal identity [309] (Fig. 4).

**Fig. 6:**
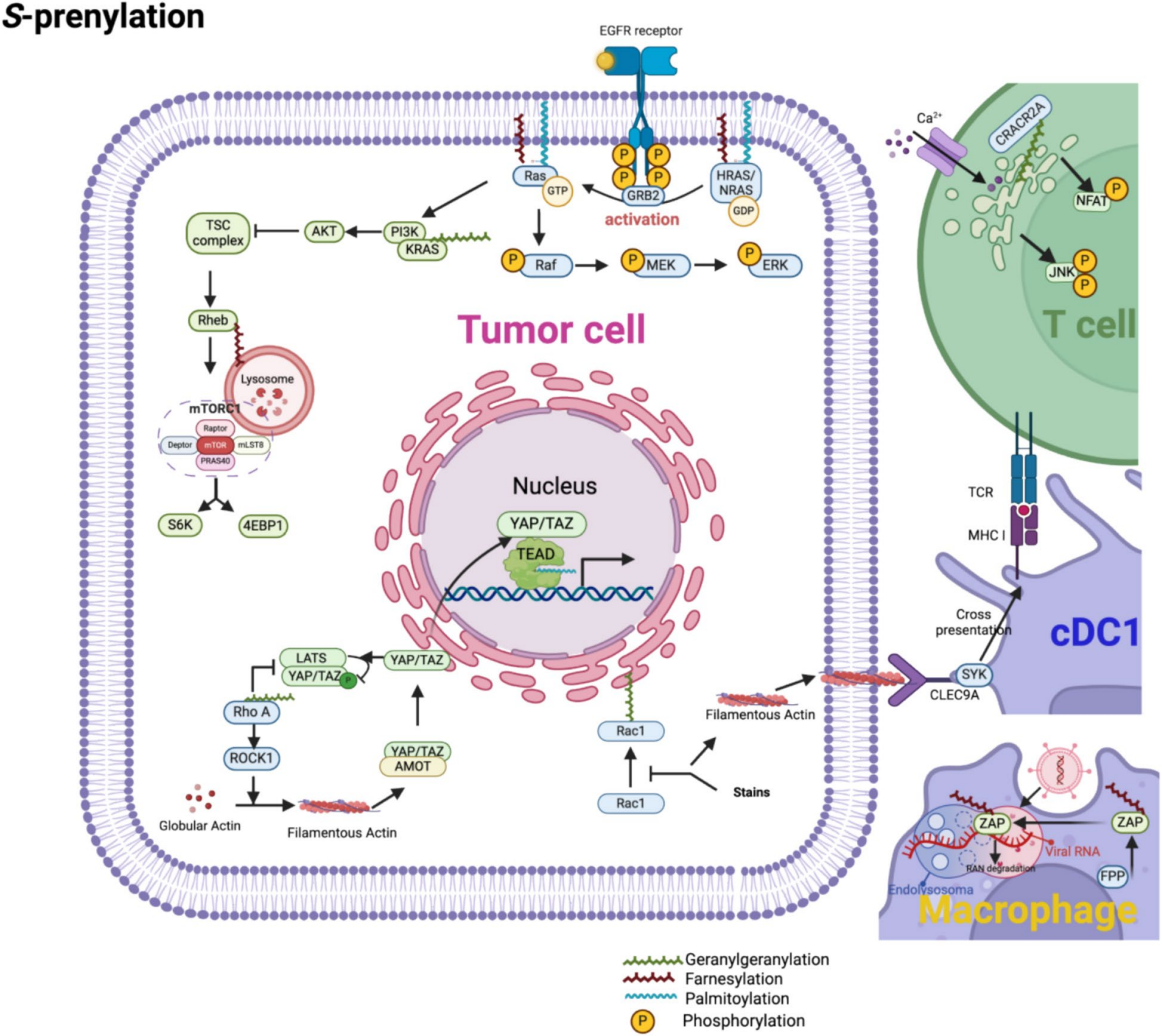
The role of *S-*prenylation within the TME. Key pathways and molecules influenced by *S-*prenylation are summarized. 1. RAS pathway: *S-*prenylation of Ras (rat sarcoma viral oncogene homolog) proteins initiates the Ras signaling cascade by promoting GDP–GTP exchange, enabling Ras translocation to the membrane, activation of the Ras–Raf–MEK–ERK (rapidly accelerated fibrosarcoma-mitogen-activated protein kinase kinase-extracellular signal-regulated kinase) pathway, and facilitating the interaction between KRAS (Kirsten rat sarcoma viral oncogene homolog) and PI3 K (phosphatidylinositol 3-kinase), which activates the PI3 K pathway, regulating modulating excessive inflammatory responses. 2. Rheb-mTORC1: *S-*prenylation of Rheb (Ras homolog enriched in brain) localizes it to the lysosomal surface, where it activates mTORC1 (mechanistic target of rapamycin complex 1). Upstream, AKT (protein kinase B) inhibits TSC1/TSC2 (tuberous sclerosis complex 1/2), preventing Rheb suppression and sustaining mTORC1 signaling, which activates S6 K (ribosomal protein S6 kinase) and 4EBP1 (eukaryotic translation initiation factor 4E-binding protein 1), promoting protein synthesis and tumor metabolism. 3. Hippo Pathway: *S-*prenylation of RhoA (Ras homolog family member A) activates YAP/TAZ (yes-associated protein/transcriptional coactivator with PDZ-binding motif) by inhibiting LATS1/2 (large tumor suppressor kinase 1/2), driving oncogenic TEAD/YAP (TEA domain transcription factor-YAP) transcription. Moreover, RhoA indirectly hyperactivates RhoA/ROCK1 (Rho-associated coiled-coil containing protein kinase 1)/actomyosin mechano-signaling to promote oncogenic TEAD/YAP transcription further. 4. Rac1: Inhibition of Rac1 (Ras-related C3 botulinum toxin substrate 1) geranylgeranylation triggers exposure of damaged filaments, recognized by CLEC9 A (C-type lectin domain family 9 A) on cDC1 s (conventional dendritic cells type 1), and cross-presentation to activate CD8⁺ T cells. 5. TCR (T-cell receptor): *S-*prenylation localizes CRACR2 A (calcium release-activated channel regulator 2 A) to the Golgi, regulating calcium influx, JNK (c-Jun N-terminal kinase) signaling, and vesicle transport, enhancing TCR activation and immune synapse formation. 6. ZAP (zinc-finger antiviral protein): Farnesylation of ZAP long isoform targets endolysosomes, facilitating viral RNA degradation and innate immunity. 4EBP1: Eukaryotic translation initiation factor 4E-binding protein 1; AKT: Protein kinase B; AMOT: Angiomotin; cDC1: Conventional dendritic cell type 1; CLEC9 A: C-type lectin domain family 9 A; CRACR2 A: Calcium release-activated channel regulator 2 A; ERK: Extracellular signal-regulated kinase; FPP: Farnesyl pyrophosphate; JNK: c-Jun N-terminal kinase; KRAS: Kirsten rat sarcoma viral oncogene homolog; LATS: Large tumor suppressor kinase; MEK: Mitogen-activated protein kinase kinase; mTORC1: Mechanistic target of rapamycin complex 1; PI3 K: Phosphatidylinositol 3-kinase; Rac1: Ras-related C3 botulinum toxin substrate 1; Rheb: Ras homolog enriched in brain; RhoA: Ras homolog family member A; ROCK1: Rho-associated coiled-coil containing protein kinase 1; S6 K: Ribosomal protein S6 kinase; TEAD: TEA domain transcription factor; TSC: Tuberous sclerosis complex; YAP/TAZ: Yes-associated protein/Transcriptional coactivator with PDZ-binding motif; ZAP: Zinc-finger antiviral protein; ZDHHC: Zinc finger DHHC-type containing protein

At the cytoskeletal level, *S-*prenylation interlocks with these lipid modifications to orchestrate cytoskeletal remodeling. The *S-*prenylation-dependent activation of RhoA and Rac1, facilitated by ICMT [310], reinforces actomyosin contractility and cell shape changes required for EMT progression and tumor metastasis [311]. In particular, TP53 mutations hyperactivate RhoA *S*-prenylation through the MVA pathway, amplifying RhoA/ROCK1/actomyosin mechano-signaling, which suppresses Lats1/2 activity and enhances TEAD/YAP transcriptional activity, reinforcing a metastatic phenotype [312–314] (Fig. 6). Beyond EMT regulation, lipidation is a central orchestrator of tumor cell movement, determining adhesion, cytoskeletal flexibility, and ECM interactions. *N-*myristoylation of MARCKS enhances fibronectin adhesion, stabilizing cell–matrix interactions [315, 316], yet paradoxically, prenylated RhoA activation simultaneously primes actin polymerization, fostering detachment and migration [313, 317]. This lipidation-driven tug-of-war enables tumor cells to finely regulate adhesion strength, balancing cell–matrix interactions with the need for invasive mobility.

At the membrane level, *S-*palmitoylation of FAK [318], a key EMT effector, facilitates its membrane localization. Meanwhile, *N-*myristoylation of p22, a Ca^2^⁺- binding protein, facilitates microtubule attachment, promoting intracellular trafficking essential for directional migration [319]. Intriguingly, the formins FMNL2 and FMNL3, which regulate spindle migration, rely on *N-*myristoylation to optimize cytoskeletal flexibility, reinforcing actin polymerization for cell shape adjustments [320, 321].

### Innate immune responses

Growing evidence suggests that the innate and adaptive immune systems contribute to tumor progression when they are present in the TME [2]. Innate immune cells, including macrophages, dendritic cells (DCs), natural killer (NK) cells, and B cells, play crucial roles in tumor immunity within the TME, the innate immune system serves as the first line of defense against pathogens through pattern recognition receptors (PRRs). PRRs detect pathogen-associated molecular patterns (PAMPs) and damage-associated molecular patterns (DAMPs). This recognition triggers the production of proinflammatory cytokines and immunomodulatory molecules. PRRs are expressed primarily in antigen-presenting cells, such as DCs and macrophages [322–324].

#### Tumor-associated macrophages (TAMs)

Macrophages are a major component of the innate immune system in the TME and can be polarized into pro-inflammatory (M1-like) or immunosuppressive (M2-like) phenotypes, influencing tumor progression. Lipidation regulating macrophage function by modulating TLR signaling, cytokine production, and phagocytosis.

Lipopolysaccharide (LPS) from gram-negative bacteria activates the Toll-like receptor 4 (TLR4) signaling pathway, which involves four adaptor molecules: myeloid differentiation primary response 88 (MyD88), MyD88 adapter-like (MAL), Toll/IL- 1 receptor domain-containing adapter inducing IFN-β (Trif), and Trif-related adapter molecule (TRAM) [325]. The *N-*myristoylation of TRAM is critical for the activation of TLR4 signaling, which, upon LPS stimulation, facilitates TRAM’s translocation to the PM and its phosphorylation by protein kinase C ε (PKCε). This leads to the recruitment of Trif, activating nuclear factor kappa B (NF-κB) and interferon regulatory factor 3 (IRF3), promoting the pro-inflammatory response [326, 327] (Fig. 4). As a myristate-binding proteinheme oxygenase (HO- 2) negatively regulates the TRAM-dependent LPS‒TLR4 pathway, modulating the intensity of the immune response [328]. Beyond TLR signaling, *N-*myristoylation of ARF1 inhibits the cyclic GMP-AMP synthase (cGAS)-STING pathway, reducing the production of type I interferons (IFN-I) and dampening antitumor immunity [223]. Meanwhile, *S-*palmitoylation of STING aggregated at the Golgi apparatus, enhance IFN-I response, which, depending on strength and duration, may either promote tumor elimination or support immune evasion [329, 330] **(**Fig. 5**)**. In contrast, *S-*palmitoylation of cGAS inhibits its binding to DNA, negatively regulating antitumor immune responses [331]. The *S-*palmitoylation of upstream proteins [332, 333] affects IFN*-*I responses, and downstream proteins depend on *S-*palmitoylation and influence antitumor immunity. Upon activation, IFN I activates the JAK-STAT pathway through the IFN*-*alpha receptor subunit (IFNAR1) to amplify immune responses [334]. Like a checkpoint, *S-*palmitoylation of IFNAR1 accelerates its degradation to prevent sustained activation and activates STAT2, indirectly affecting the transcription of STAT1 and IFN*-*α [335]. *S-*palmitoylation also affects the membrane localization of GPCR family members(e.g., CCR5 [336] and Rac1 [337]), influencing macrophage responses to inflammatory chemical signals. *S-*prenylation is also essential for maintaining macrophages' normal anti-inflammatory functions. By promoting the interaction between KRAS and PI3 K, the PI3 K pathway is activated, which regulates TLR signaling and moderates excessive inflammatory responses [338]. For the long isoform of the ZAP antiviral protein, farnesylation is essential for its targeting endolysosomes, thereby increasing its antiviral activity [339] (Fig. 6).

Phagocytosis, another central function of macrophages, is also influenced by lipidation. *S-*palmitoylation enhances Fcγ receptor-mediated phagocytosis in macrophages, with ZDHHC6-dependent lipidation increasing the efficiency of receptor clustering [340]. Similarly, CD36, a scavenger receptor involved in lipid uptake, relies on *S-*palmitoylation for its localization, further supporting macrophage activation [341]. Moreover, bacterial-sensing receptors such as nucleotide-binding oligomerization domain (NOD1/2) receptors require *S-*palmitoylation for activation, ensuring an effective immune response to microbial and damage-associated signals [342, 343]. Geranylgeranylation is critical for cytoskeletal dynamics, insufficient GGPP impairs the ability of monocytes and macrophages to respond to chemokines [344].

#### DCs

The impact of lipidation extends beyond macrophages to DCs, which act as crucial antigen-presenting cells, bridging the innate and adaptive immune response. *N-*myristoylation regulates endocytosis in DCs, as demonstrated by the HIV- 1 protein Nef, which prevents DC internalization of antigens by upregulating DC-SIGN expression, disrupting antigen processing and immune synapse formation [345, 346]. Similarly, *S-*palmitoylation enhances antigen uptake and presentation by facilitating the internalization of palmitoylated peptides, which are then processed and loaded onto major histocompatibility complex(MHC) I molecules, accelerating the immune activation process, making *S-*palmitoylation a powerful tool for antigen enrichment [347]. Moreover, *S-*palmitoylation modulates the production of IFN*-*α by activating interferon regulatory factor 7, ensuring proper DC inflammatory responses [348]. Blocking TLR2 *S-*palmitoylation inhibits cell surface expression and diminishes inflammatory responses to microbial ligands [349]. Structural organization within DCs is also lipidation-dependent; cDC1 s utilize their unique cross-presentation ability to degrade engulfed extracellular proteins and present them through MHC I molecules, activating CD8⁺ T cells and initiating antitumor immune responses. Geranylgeranylated Rac1 is necessary for actin filament reorganization, these proteins are then recognized by CLEC9 A (a C-type lectin receptor) on cDC1s, further activating CD8⁺ T cells and strengthening antitumor immunity (Fig. 6) [275, 350].

#### NK cells

**Fig. 7:**
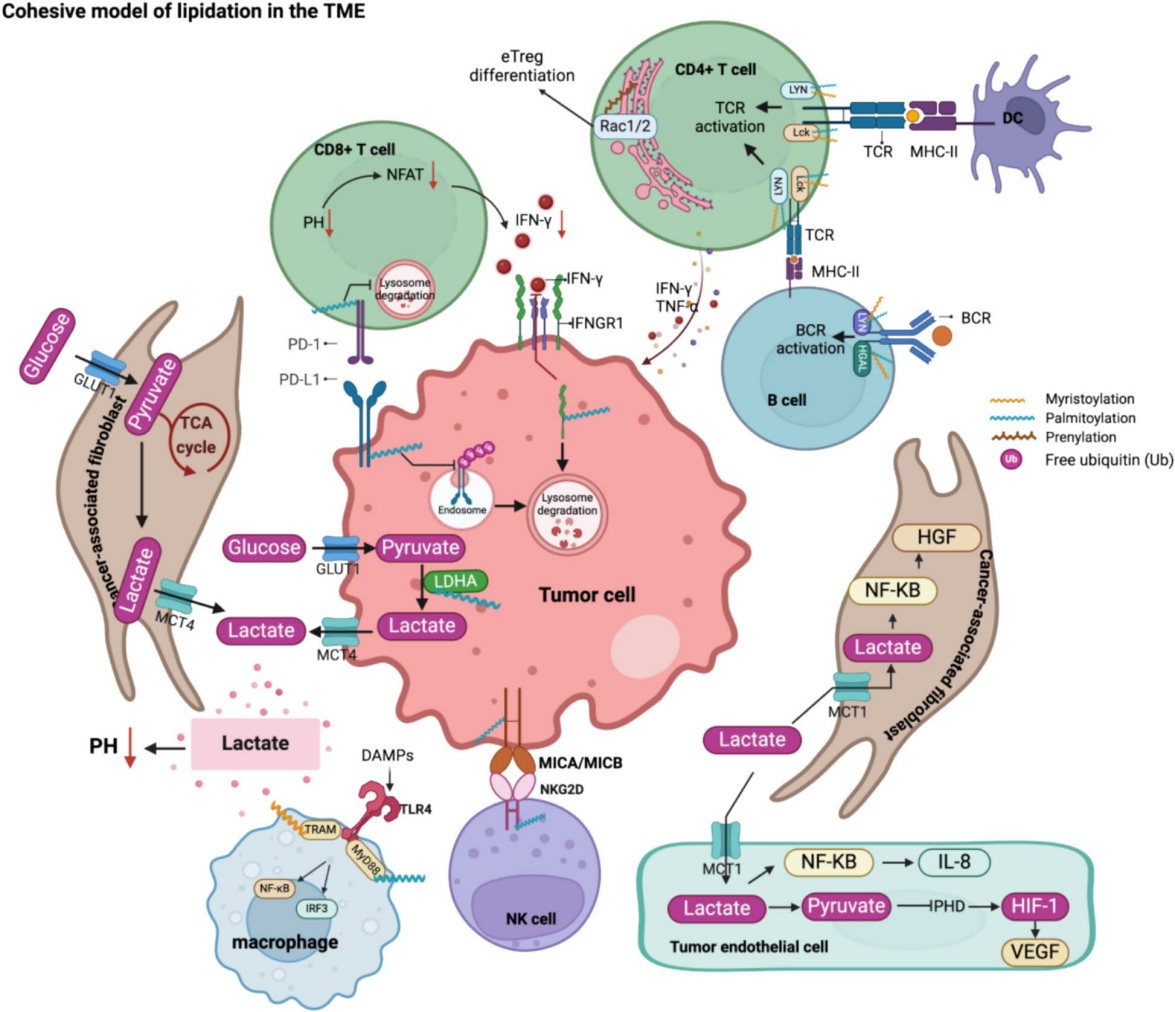
Cohesive model of lipidation in the TME. 1. TCR Signaling: *N-*myristoylation and *S-*palmitoylation regulate T-cell receptor (TCR) signaling by ensuring membrane localization and activation of Lck and Fyn in CD4⁺ T cells. Upon TCR activation, CD4⁺ T cells release cytokines such as IFN-γ and TNF-α, modulating immune responses and shaping the TME. 2. BCR Signaling: In B cells, *N-*myristoylation and *S-*palmitoylation ensuring the proper membrane anchoring of Lyn and HGAL. Following BCR activation, B cells facilitate antigen presentation via major histocompatibility complex class II (MHC II) through human leukocyte antigen HLA-DM/HLA-DO, enhancing CD4⁺ T-cell activation and adaptive immunity. 3. PD- 1/PD-L1: *S-*palmitoylation stabilizes PD-L1 and PD- 1, preventing their lysosomal degradation. This prolongs PD- 1/PD-L1 interactions, thereby inhibiting TCR activation, reducing T-cell cytotoxicity, and allowing tumors to evade immune surveillance. 4. IFN-γ Signaling: *S-*palmitoylation of interferon gamma receptor 1 (IFNGR1)serves as a lysosomal sorting signal, reducing IFN-γ responsiveness and impairing MHC-I upregulation, which dampens CD8⁺ T-cell activation. Additionally, excessive lactate production by tumor cells acidifies the TME, lowering pH, suppressing NFAT, and reducing IFN-γ secretion, further contributing to immune evasion. 5. Lactate Metabolism: *S-*palmitoylation of lactate dehydrogenase A (LDHA) enhances glycolytic flux, increasing lactate accumulation in the TME. Lactate acidifies the TME, suppressing NFAT,thereby impairing T-cell function. Cancer-associated fibroblasts (CAFs) and tumor endothelial cells uptake lactate via monocarboxylate transporter 1 (MCT1) and utilize it as an energy source, and activate the NF-κB/HGF signaling and NF-κB/IL- 8 pathway, respectively. while glycolytic tumor cells and CAFs export lactate via MCT4, forming a metabolic symbiosis. Lactate also stabilizes HIF- 1α, inducing VEGF production, further supporting tumor growth and vascularization. 6. TLR4 in Macrophages: Upon recognition of damage-associated molecular patterns (DAMPs), Toll-like receptor 4 (TLR4) in macrophages is activated. *S-*palmitoylation of MyD88 by ZDHHC6 is required for the recruitment of TLR signaling complexes, driving NF-κB activation and proinflammatory cytokine release. *N-*myristoylation of TRAM directs its localization to plasma membrane microdomains, where PKCε phosphorylation facilitates TRIF-mediated IFN-I signaling in response to TLR4 activation, shaping macrophage polarization within the TME. 7. NK Cell: Natural killer (NK) cells rely on *S-*palmitoylation to enhance receptor-ligand clustering within lipid rafts, such as NKG2D-MICA/MICB interactions, thereby optimizing tumor cell recognitionand cytotoxicity. TCR: T-cell receptor; BCR: B-cell receptor; Lck: Lymphocyte-specific protein tyrosine kinase; Fyn: Proto-oncogene tyrosine-protein kinase Fyn; Lyn: Lck/Yes-related novel protein tyrosine kinase; CD4⁺ T cells: Cluster of differentiation 4-positive T cells; NFAT: Nuclear factor of activated T cells; TNF-α: Tumor necrosis factor-alpha; IFN-γ: Interferon gamma; PD- 1: Programmed cell death protein 1; PD-L1: Programmed death-ligand 1; MHC II: Major histocompatibility complex class II; LDHA: Lactate dehydrogenase A; MCT1: Monocarboxylate transporter 1; MCT4: Monocarboxylate transporter 4; HIF- 1α: Hypoxia-inducible factor 1-alpha; VEGF: Vascular endothelial growth factor; NF-κB: Nuclear factor kappa-light-chain-enhancer of activated B cells; HGF: Hepatocyte growth factor; TLR4: Toll-like receptor 4; TRAM: TRIF-related adaptor molecule; MyD88: Myeloid differentiation primary response 88; IRF3: Interferon regulatory factor 3; DAMPs: Damage-associated molecular patterns; NK: Natural killer cells; NKG2D: Natural killer group 2D receptor; MICA/MICB: MHC class I polypeptide-related sequence A/B; PHD: Prolyl hydroxylases

The function of NK cells, which provide rapid cytotoxic responses to tumor cells, is also regulated by lipidation. *S-*palmitoylation enhances the clustering of NK receptors such as NKG2D and 2B4 with their ligands (MICA/MICB) within cholesterol-rich microdomains, optimizing NK cell activation and tumor cell recognition [351, 352] (Fig. 7). Additionally, *S-*palmitoylation ensures syntaxin 11 localization at immune synapses, maintaining its interaction with Munc18 - 2 and promoting lysosome exocytosis, thereby enhancing NK cell-mediated cytotoxicity [353].

### Adaptive immune responses

#### B cells

B cells, essential component of the immune response, rely on lipidation for the proper function of B-cell receptor (BCR) signaling. N-myristoylation and *S-*palmitoylation of Lyn and human germinal center-associated protein (HGAL) stabilizes these proteins within lipid raft microdomains, enhancing their interactions with downstream effectors [354–356] (Fig. 4). This dual lipidation-dependent clustering facilitates the phosphorylation of immunoreceptor tyrosine-based activation motifs (ITAMs) by Lyn, activating spleen tyrosine kinase (SYK), Bruton’s tyrosine kinase (BTK), and phospholipase Cγ (PLCγ), which amplify downstream signals via the NF-κB, PI3 K/AKT, MAPK/extracellular signal-related kinase (ERK), and nuclear factor of activated T cells (NFATs) pathways [354–356]. In parallel, *S-*palmitoylation enhances BCR signaling by promoting the aggregation of BCR complexes with CD19/CD21/CD81 within lipid raft domains, prolonging signal transduction and increasing B-cell activation [357]. However, during B-cell apoptosis, caspase- 3/7 mediated cleavage of Lyn removes its N-myristoylation and *S-*palmitoylation sites, leading to its delocalization from the membrane, thereby preventing c-Myc regulation and suppressing B-cell survival [358]. Pan-NMT inhibitors block the *N-*myristoylation of Lyn, HGAL, and ARF1, accelerating their degradation and effectively inhibiting BCR signal transduction [354].

#### T cells

T-cell development and function are critically dependent on lipidation. In the TME, disruptions in *S-*palmitoylation impair key signaling proteins, such as the linker for activation of T cells (LAT), leading to T-cell anergy and immune dysfunction. Restoring LAT *S-*palmitoylation, alongside Lck expression, has been suggested as a strategy to reverse T-cell exhaustion and enhance antitumor responses [359]. Similarly, N-myristoylation is essential for maintaining T-cell homeostasis, as its deficiency results in aberrant T-cell development due to impaired Lck function, leading to a reduced number of CD4⁺ and CD8⁺ single-positive T cells in lymphoid organs [360]. Beyond development, NMT1 deficiency disrupts metabolic signaling by suppressing the AMPK pathway while hyperactivating TORC1, skewing T-cell differentiation toward proinflammatory TH1 and TH17 subsets and driving excessive IFN-γ and IL- 17 production, which may exacerbate autoimmune responses [361]. Interestingly, γδ T cells, which serve as immune sentinels against tumors, exhibit a unique independence from thymic N-myristoylation, though its inhibition disrupts the calcineurin–NFAT pathway and paradoxically enhances IFN-γ secretion [362, 363]. The depletion of GGPP induces stress responses in monocytes, triggering caspase- 1 activation and IL- 18 release, which in turn drives γδ T-cell activation [364].

T-cell activation is precisely regulated by *S-*palmitoylation and *N-*myristoylation, which work in concert to stabilize key immune receptors and fine-tune TCR signal transduction (Fig. 3). *S-*palmitoylation prevents the degradation of CD80, maintaining its stability at the immune synapse and promoting costimulatory interactions essential for full T-cell activation [365]. Additionally, it governs the localization of CD4, CD8, and LAT in cholesterol-rich membrane microdomains, ensuring that TCR signaling components are properly assembled [366–368]. Following TCR engagement, *S-*palmitoylation drives the phase separation of Grb2, Gads, SOS, and Slp76, leading to the formation of distinct signaling clusters that amplify downstream activation cascades [369]. Crucially, the activation of the TCR complex depends on the lipidation of Src family kinases, particularly Lck and Fyn, which require both *N*-myristoylation and *S-*palmitoylation for proper localization and function [370, 371]. Lck, a central mediator of TCR signaling, relies on these dual lipid modifications to interact with ZAP70 and PLC-γ1, initiating phosphorylation cascades that reinforce T-cell activation [372]. Likewise, Fyn undergoes dual lipidation to enhance ITAM phosphorylation on CD3ζ, facilitating the recruitment of Lck and amplifying TCR signaling [373, 374]. Once recruited to the TCR complex, ZAP70 phosphorylates LAT and SLP76, a process that is critically dependent on *S-*palmitoylation to stabilize these proteins within signaling microdomains [375, 376]. The transport of LAT-containing vesicles to the immune synapse is further regulated by ZDHHC18, highlighting a lipidation-dependent mechanism that sustains TCR responses [377]. While LAT has long been associated with lipid raft domains, emerging evidence suggests that its stability and plasma membrane anchoring are primarily dictated by *S-*palmitoylation, serving as a sorting signal for its localization [378, 379]. This process is linked to CD59, which appears to assist in the transfer of palmitoyl groups to LAT [380, 381], and prevent LAT degradation, thus prolonging TCR signaling duration and ensuring sustained immune activation [382, 383].

The role of *S-*prenylation in TCR signaling is further exemplified by CRACR2 A, a Rab GTPase that requires *S-*prenylation for its proper localization near the Golgi apparatus, ensuring effective control of calcium influx, JNK signaling, and vesicle trafficking [384]. These processes collectively support immune synapse formation and enhance the precision of T-cell activation (Fig. 6).

#### Immune checkpoints

PD- 1 is expressed on activated T cells, while PD-L1 is found on tumor and immune cells, where their interaction modulates immune responses and contributes to immune evasion [385] (Fig. 5). In tumor cells, *S-*palmitoylation of PD-L1 acts as a critical molecular switch regulating its stability and function. DHHC3-mediated *S-*palmitoylation of PD-L1 prevents its monoubiquitination, blocking its degradation through the endosomal sorting complex required for transport pathway and lysosomal degradation [21]. This enhances PD-L1 interaction with PD- 1, suppressing T-cell antitumor responses and promoting immune evasion. Inhibition of *S-*palmitoylation promotes PD-L1 degradation and restores T-cell activity, suggesting a potential therapeutic approach to counter PD-L1-mediated immune suppression [21].

Although DHHC3 mediates PD-L1 *S-*palmitoylation in colorectal cancer (CRC) cells, ZDHHC9 is identified as the primary *S-*palmitoylation enzyme for PD-L1 in other cancers, lung adenocarcinoma [268], PDAC [386] and BrCa [387], ZDHHC9-mediated *S-*palmitoylation stabilizes PD-L1, promoting immune evasion. A lack of ZDHHC9 shifts the TME from an immunosuppressive (“cold”) state to a proinflammatory (“hot”) state, primarily through CD8 + T-cell-dependent mechanisms, increasing the sensitivity of tumors to anti-PD-L1 immunotherapies [386]. Increased *S-*palmitoylation of PD-L1 is associated with chemotherapy resistance in tumor cells [388].

Similarly, *S-*palmitoylation of PD- 1, mediated by ZDHHC9, prevents its lysosomal degradation and enhances its stability, contributing to immune evasion. Palmitoylated PD- 1 also activates the mTOR pathway, promoting tumor cell proliferation. In three-dimensional culture systems, targeting PD- 1 *S-*palmitoylation has shown significant antitumor effects [138]. By inhibiting PD- 1 *S-*palmitoylation, the accumulation of PD- 1 on the tumor cell surface is reduced, thereby enhancing T-cell-mediated tumor immune responses. Targeting PD- 1 and PD-L1 *S-*palmitoylation, combined with immune checkpoint inhibitors or CAR-T cell therapies, may help overcome the existing clinical drug resistance. T-cell immunoglobulin and mucin domain-containing protein 3 (TIM- 3) is an emerging immune checkpoint, and its high expression exacerbates dysfunction and exhaustion in CD8 + T cells and NK cells while blocking TIM- 3 enhances their antitumor function [389]. The *S-*palmitoylation of TIM- 3 is regulated by DHHC9, which inhibits the interaction between TIM- 3 and HRD1, preventing its ubiquitination and degradation, thus stabilizing the TIM- 3 protein [390].

Thus, ZDHHC9 plays a crucial role in the tumor immune microenvironment, particularly in immune checkpoint regulation. Therefore, inhibiting ZDHHC9 emerges as a promising anticancer strategy. Given the emerging resistance to current immune checkpoint inhibitors in clinical settings, developing inhibitors targeting *S-*palmitoylation, such as ZDHHC9 selective inhibitors, is particularly important, and preclinical studies have demonstrated their efficacy [138]. Furthermore, the combination of immune checkpoint inhibitors with CAR-T therapy has shown significant therapeutic efficacy in recent studies [391], in a study of 11 mesothelioma patients who underwent preconditioning with cyclophosphamide, followed by a single dose of mesothelin-targeted CAR-T cells and at least three doses of anti-PD- 1 therapy, two patients achieved a 72% response rate with complete metabolic responses [392], further validating the potential of this combinatorial approach. Notably, research has also revealed surprising effects from the combination of ZDHHC9 inhibition and CAR-T therapy, such as the attenuation of CAR-T cell exhaustion upon DHHC9 knockdown and the acceleration of TIM- 3 degradation through a TIM- 3 *S-*palmitoylation peptide inhibitor, which enhances the antitumor immune response mediated by CAR-T cells and NK cells [390].

### Cancer-associated fibroblasts

Cancer-associated fibroblasts(CAFs) in the TME are metabolically reprogrammed to support tumor growth by supplying fatty acids and modulating lactate metabolism. Lipidation, particularly *S-*palmitoylation, regulates multiple aspects of fatty acid uptake, storage, and metabolic signaling in these stromal cells, influencing tumor progression and immune evasion. regulate many aspects of fatty acid uptake and storage in these stromal cells.

A key regulator of fatty acid metabolism in CAFs is CD36, a membrane receptor that mediates the uptake of long-chain fatty acids. CD36 undergoes *S-*palmitoylation on intracellular cysteine residues, which is essential for its efficient localization to the PM and lipid rafts. In these membrane microdomains, palmitoylated CD36 forms complexes with Src-family kinases like Fyn and Lyn, facilitating fatty acid internalization and metabolic signaling [393]. Disrupting CD36 *S-*palmitoylation causes its retention in intracellular compartments, reduces fatty acid uptake, and attenuates downstream metabolic signaling, such as AMPK inhibition. TAMs [394] and CAFs [395] both express CD36, suggesting that heightened CD36 *S-*palmitoylation in these stromal cells promotes fatty acid influx for metabolic adaptation. Additionally, CD36 mediates oxidized LDL uptake-dependent macrophage migration inhibitory factor (MIF) expression, which recruits CD33 + myeloid-derived suppressor cells (MDSCs) and contributes to immune suppression and immunotherapy resistance [395].

In the Reverse Warburg Effect, glycolytic CAFs serve as the primary lactate producers in the TME, exporting lactate via monocarboxylate transporter 4 (MCT4), which is then taken up by tumor cells through MCT1. This lactate shuttle fuels tumor metabolism and enhances invasion by activating the TGF-β1/p38 MAPK/MMP2/9 signaling pathway [396]. 

Beyond metabolism, MAGUK p55 subfamily member (Mpp7) is a membrane-palmitoylated protein associated with CAFs that influences cancer progression by regulating the Rap1 signaling pathway [397]. Moreover, Mpp6 mediates the protumorigenic activity of serum amyloid A3 and stimulates the growth of pancreatic cancer cells [398]. In addition, *S-*palmitoylation inhibits receptor tyrosine kinase-mediated MAPK pathways by anchoring Spry proteins, thereby inhibiting cell proliferation and differentiation induced by FGF and VEGF and limiting the excessive activation of fibroblasts [399]. *S-*palmitoylation also regulates the membrane localization of the Rho family GTPase Wrch- 1, which promotes anchorage-dependent growth transformation in fibroblasts [400]. It induces large-scale membrane restructuring and massive endocytosis under mitochondrial stress conditions, thus maintaining membrane integrity and function [401].

### Cancer-associated endothelial cells

eNOS is a central mediator of pro-angiogenic signaling, as it produces nitric oxide (NO) to induce vasodilation and support vessel sprouting [402–406]. eNOS is one of the best-characterized dually lipid-modified proteins: it undergoes co-translational *N-*myristoylation and post-translational *S-*palmitoylation on its N-terminus, these modifications target eNOS to the Golgi and then to plasma membrane caveolae in endothelial cells (ECs) [407, 408]. *S-*palmitoylation of eNOS at two N-terminal cysteines (Cys15 and Cys26) is mediated by DHHC21 and is reversible [409]. Proper localization of eNOS in caveolae (which are rich in the palmitoylated protein caveolin- 1 [410]) is required for its optimal activation by calcium-CaM [411] and Akt signaling [412]. Mutations that prevent eNOS N-myristoylation [413] or *S-*palmitoylation [414] cause eNOS to mislocalize to the cytosol and drastically reduce NO production. This leads to impaired angiogenic responses since NO is a pro-survival and pro-migratory signal for ECs. Studies have shown that agonist such as bradykinin stimulation of ECs triggers dynamic depalmitoylation of eNOS, causing it to temporarily leave caveolae and generate NO in other membrane compartments [415]. This cycle of *S-*palmitoylation/depalmitoylation is thought to fine-tune NO release during blood vessel remodeling. The importance of eNOS coupling further underscores by metabolic *S-*palmitoylation: ECs rely on endothelial cell-intrinsic FASN to supply palmitate for eNOS modification. When FASN is knocked out in endothelium, eNOS *S-*palmitoylation is lost, and reparative angiogenesis is impaired [416]. Thus, maintaining eNOS lipidation is a crucial aspect of tumor angiogenesis.

Another component is VEGF signaling. The primary VEGF receptor on endothelium, VEGFR1, which can act as a decoy or modulator of VEGF, exists in a membrane-bound form (mVEGFR1) that is palmitoylated, which serves as a “binary switch” for its stability: palmitoylated VEGFR1 is retained on the PM and recycled, whereas depalmitoylation targets VEGFR1 for internalization and lysosomal degradation [417]. Rab27a, a prenylated small GTPase, was identified as a critical upstream regulator of this process which help Traffick the *S-*palmitoylation enzymes [417]. Inhibition of FTase reduces the expression of hypoxia-inducible factor 1-alpha (HIF1α), prevents the binding of HIF1α to heat shock protein 90, and decreases the production of VEGF, thereby inhibiting angiogenic activities [418]. Additionally, other endothelial proteins required for vessel integrity are palmitoylated, for example, platelet endothelial cell adhesion molecule 1 is *S-*palmitoylated, which promotes its cell-surface localization in ECs [419]. Aberrant *S-*palmitoylation of such adhesion molecules could lead to the dysfunctional, leaky vessels characteristic of tumors. Beyond adhesion molecules, *S-*palmitoylation also influences endothelial motility and angiogenesis. The *S-*palmitoylation of RhoJ, regulated in a GLUL-dependent manner, enhances EC motility and promotes angiogenesis [420]. Similarly, the PI3 K–AKT–mTOR pathway, which drives angiogenesis and cell proliferation in clear cell renal cell carcinoma, can be activated through the *S-*palmitoylation of AGK [175]. In contrast, *S-*palmitoylation can also exert anti-angiogenic effects. For example, activation of the Src/p53 signaling pathway via *S-*palmitoylation of KAI1 inhibits angiogenesis [421].

For new blood vessels to form and infiltrate a growing tumor, endothelial cells must proliferate, migrate, and invade the surrounding matrix. These processes are driven by cytoskeletal remodeling and cell polarity pathways that heavily rely on prenylated small GTPases like Rho, Rac, and Cdc42 [422]. Active Rac1 drives lamellipodia formation for cell migration [423], while RhoA and RhoB influences stress fibers [424] and cell contraction [425]. In tumor angiogenesis, excessive Rho/Rac signaling can lead to abnormal vessel structure and hyper-permeability, aiding tumor cell intravasation. Pharmacological studies with statins which block the MVA pathway and thus *S-*prenylation illustrate the role of these GTPases: simvastatin was found to interfere with angiogenic sprouting by preventing Rho geranylgeranylation, thereby inhibiting Rho-mediated actin changes, effects reversible by adding back geranylgeranyl pyrophosphate [422].

### Integrate role of lipidation in the TME

Protein lipidation does not act in isolation; rather, it forms an intricate signaling network that unifies immune evasion [21], metabolic reprogramming [426], and angiogenesis [416] within the TME. Tumor cells exploit lipidation to modulate immune signaling and evade immune surveillance [427]; simultaneously, immune and stromal cells leverage lipidation to respond dynamically to tumor-derived metabolic cues, such as lactate [428, 429], maintaining a complex and delicately balanced TME (Fig. 7).

Lipidation critically regulates TCR and BCR signaling by ensuring the proper membrane localization and activation of Src-family kinases (SFKs), such as Lck [372] and Fyn [373, 374] in T cells, and Lyn [354–356] in B cells. This lipidation-driven membrane localization amplifies downstream signaling cascades involving ZAP70 [372], LAT [376] in TCR signaling, and SYK, BTK, and PLCγ2 [354–356] in BCR signaling, thereby activating pathways like NF-κB and NFAT [354–356]. Indeed, BCR-mediated antigen recognition is not only the initiating signal for intracellular signaling in B cells but also regulates MHC II antigen presentation through human leucocyte antigen HLA-DM/HLA-DO, thereby enhancing T cell activation [430]. After TCR activation, cytotoxic T lymphocytes form an immune synapse with tumor cells, leading to the release of perforin, which creates membrane pores, allowing granzymes to enter and trigger tumor cell apoptosis [431]. However, tumor cells exploit lipidation to promote immune evasion. ZDHHC9-mediated *S-*palmitoylation stabilizes PD- 1 [138], which recruits Src homology region 2 domain-containing phosphatases. This inhibits TCR and CD28 signaling, weakening T cell activation, antigen recognition, and cytotoxic function, thereby sustaining T cell exhaustion and allowing tumors to escape immune surveillance [432].

In addition, tumor cells drive the differentiation of regulatory B cells (Bregs), which secrete IL- 10 and TGF-β while suppressing IFN-γ, TNF, and MCP- 1 production, this shifts the TME toward immunosuppression, inhibiting CD8⁺ and CD4⁺ T cell activation and impairing TCR-mediated cytotoxic responses [433, 434]. Bregs promote Treg differentiation [435] which the immune suppression effect was further reinforced. Within Treg, FTase promotes the maintenance of effector Tregs, GGTase I is a rheostat for TCR-dependent transcriptional programming and Rac-mediated signaling, establishing eTreg cell differentiation and immune tolerance [436].

Conversely, lipidation can enhance antitumor responses by optimizing innate immune receptor clustering. NK cells rely on *S-*palmitoylation to enhance receptor-ligand interactions within lipid rafts, such as NKG2D-MICA/MICB and 2B4-CD48, facilitating tumor cell recognition and cytotoxicity [351, 352]. *S-*palmitoylation of MyD88 by ZDHHC6 is required to recruit TLR complexes, driving NF-κB activation and proinflammatory cytokine release [437]. N-myristoylation of TRAM directs its localization to PM microdomains, which phosphorylated by PKCε facilitates TRIF-mediated IFN-I signaling in response to TLR4 activation [326, 327]. However, *S-*palmitoylation of IFNGR1 serving as a lysosomal sorting signal [427], which is driven by receptor degradation, reduces interferon responsiveness, diminishing antigen presentation and enabling immune escape and suppressing MHC class I upregulation, allowing tumor cells to escape CD8⁺ T-cell-mediated cytotoxicity [427].

The TME is characterized by metabolic reprograming, where lipidation and lactate metabolism converge to regulate cellular fitness. Palmitoylation of lactate LDHA enhances glycolytic flux, leading to lactate accumulation, a hallmark of the Warburg effect [16, 428]. This lactate surplus acidifies the TME, impairing T cell activation by inhibiting signaling pathways such as NFAT, p38, and JNK [438], while simultaneously stabilizing HIF1α [439], a master regulator of tumor metabolic adaptation. Beyond acidification, lactate acts as an intercellular metabolite, fueling metabolic coupling among tumor, stromal, and immune cells. Well-oxygenated tumor cells and CAFs internalize lactate via MCT1, utilizing it as an energy source while freeing glucose for hypoxic tumor regions—a process termed the "reverse Warburg effect" [429]. Lipidation contributes to this metabolic reprogramming by modulating key metabolic enzymes, supporting tumor proliferation and angiogenesis.

Simultaneously, lactate modifies immune signaling via G-protein coupled receptor 81 (GPR81), leading to cAMP suppression and PD-L1 upregulation, further reinforcing immune evasion [440]. DCs express GPR81, whose lactate-mediated activation suppresses the cell-surface expression of MHC-II, thereby compromising the ability of DCs to present tumor-cell antigens to T cells [441]. In cancer associate macrophages, this can shift them to an M2-like state that supports tumor growth [442]. This cross-regulation ensures a coordinated immune-metabolic landscape.

Crucially, lactate’s effects extend beyond immune modulation. Elevated lactate stabilizes HIF1α under normoxic conditions, promoting VEGF production [443]. In ECs, lactate uptake via endothelial MCT1 activates NF-κB/IL- 8 signaling, driving migration, vessel formation, and angiogenesis [444]. Simultaneously, lactate-driven metabolic stress triggers CAF activation through mitochondrial dysfunction, mitophagy, and subsequent secretion of growth factors such as hepatocyte growth factor(HGF) [445], promoting therapy resistance.

In essence, protein lipidation serves as a molecular currency traded between tumor and its microenvironment – it anchors immunomodulatory synapses, drives metabolic coupling, and tunes angiogenic signals in a unified, TME-wide manner.

## Therapeutic implications and drug development

Developing lipidation-targeting drugs presents unique challenges in clinical trial design, largely due to the broad impact of lipidation on multiple signaling pathways. A key hurdle is patient selection, as these agents influence fundamental protein modifications rather than single oncogenic drivers. Early-phase clinical trials must adopt biomarker-driven strategies, selecting patients based on lipidation profiles (e.g., high NMT1 expression, low NMT2 expression) to ensure therapeutic efficacy [446].

Another significant challenge is compensatory signaling, which can undermine treatment efficacy. Lessons from early FTI trials highlight the importance of pathway redundancy, as blocking farnesylation alone was ineffective unless targeting HRAS-mutant tumors [447]. Regulators now emphasize the need for trial designs that account for alternative modifications, such as KRAS switching to geranylgeranylation when farnesylation is inhibited [49]. To demonstrate target engagement, patient tumor samples should be analyzed in real-time.

Toxicity remains a key concern, given that lipidation is a fundamental cellular process. However, due to the innovative nature of lipidation-targeting therapies, several first-in-class agents, such as NMT inhibitor PCLX- 001 [448] and TEAD *S-*palmitoylation inhibitor ISM6331 [142], received fast track or orphan drug designations to expedite development. Early-phase clinical trials should begin with conservative dosing, escalating gradually while closely monitoring adverse events and organ function (e.g., liver enzymes, bone marrow counts) on a weekly basis.

AI is increasingly leveraged in biomarker analysis, drug candidate selection, and safety monitoring, improving target specificity while reducing off-target toxicity. Recent AI-driven models have refined the development of lipidation inhibitors, such as ISM6331, by predicting binding affinities and optimizing molecular selectivity [142, 449]. However, the ethical challenges associated with AI-driven drug development—such as data privacy, cybersecurity risks, and regulatory gaps—necessitate clear guidelines and informed consent protocols [450, 451].

For lipidation-targeting therapies to be widely accessible, clinical trials must eliminate socioeconomic biases by providing free or subsidized companion diagnostics for key biomarkers (e.g., ZDHHC9/20 status). Diverse patient enrollment and long-term follow-up are essential to evaluate therapeutic outcomes and identify late-onset toxicities. Ultimately, the integration of real-time safety monitoring, validated biomarkers, and adaptive dose modifications will be crucial to balancing therapeutic efficacy with patient safety.

### *N*-myristoylation

As we gain a clearer understanding of the effects of *N-*myristoylation on TME, we list the targets of *N-*myristoylation in the TME and its associated functions (Table 2) to facilitate a better understanding of its role. However, there are still several challenges to translating it into clinical therapy. First, the structural similarity between NMT1 and NMT2 increases the complexity of drug design due to the functional inconsistency of both NMT1/2. However, there are no well-established NMT-related targets as biological markers for risk stratification of patients, and some potential biomarkers are emerging, such as NMT1, which is highly expressed in a variety of solid tumors [19, 154–158], and its expression level is positively correlated with disease aggressiveness. For example, in lung cancer, NMT1 mRNA levels were 3.5-fold elevated in advanced (stage IV) patients compared to healthy individuals, suggesting that it may drive malignant transformation [452]. More importantly, large sample studies (e.g., 301 cases of hepatocellular carcinoma [159] and 706 cases of BrCa [453]) consistently showed that NMT1 high-expression patients had significantly shorter overall survival and were strongly associated with poor indicators such as high tumor grade and high proliferation index, whereas NMT2 was low-expressed or functionally unassociated in most tumors [454]. These evidences consistently support NMT1 as a prognostic biomarker across cancer types and a potential therapeutic target. Detection of these molecular markers in circulating tumor cells or exosomes using liquid biopsy techniques allows dynamic monitoring of therapeutic response [455]. Given the critical role of NMT in normal cells, extensive long-term toxicity studies may be required prior to the approval of NMT inhibitors.

**Table 1:** The summary of dual lipidation of target proteins

Lipidation Types	Proteins	Crosstalk Mechanisms	Functional Impact	References
*N-*myristoylation& *S-*palmitoylation	Gai 1	*N-*myristoylation favors Gαi1 protein localization to ordered lamellar membranes, and *S-*palmitoylation increases its affinity to lamellar domains with a negative charge	The reversible *S-*palmitoylation of Gαi1 functions as a molecular switch that governs its localization and mobility within the membrane, promoting its engagement with a diverse range of signaling partners	[84]
*N-*myristoylation& *S-*palmitoylation	FRS 2α	*N-*myristoylation is required before *S-*palmitoylation of FRS2α, with both modifications promoting its enrichment and reorganization within membrane rafts	This coupled modification enhances FRS2α's ability to engage in downstream signaling via the PI3 K/AKT and MAPK pathways, with *S-*palmitoylation playing a critical role in the reversible regulation of these processes	[82]
*N-*myristoylation& *S-*palmitoylation	CLMB	*N-*myristoylation is necessary for *S-*palmitoylation to occur properly; both lipid modifications ensure proper membrane targeting and stability of CLMB	*N-*myristoylation and *S-*palmitoylation enhance the affinity of CLMB for calcineurin, promoting its localization to the membrane	[456]
*N-*myristoylation& *S-*palmitoylation	Fyn	*N-*myristoylation of Fyn is a prerequisite for dynamic *S-*palmitoylation to occur	This coupled modification of Fyn ensures its proper localization to the membrane rafts, enhancing the phosphorylation of ITAMs on the CD3ζ chain. This, in turn, activates Lck and propagates T-cell signal transduction	[373, 374]
*N-*myristoylation& *S-*palmitoylation	LAMTOR1	*N-*myristoylation of LAMTOR1 is a prerequisite for dynamic *S-*palmitoylation to occur	*N-*myristoylation and *S-*palmitoylation of LAMTOR1 are necessary for stable lysosomal localization and mTORC1 activity	[83]
*N-*myristoylation& *S-*palmitoylation	Lyn	Lyn's N-myristoylation at Glycine- 2 and subsequent *S-*palmitoylation at Cysteine- 3 are essential for its association with the plasma membrane	This coupled modification ensures proper chromosome segregation throughout the cell cycle, ultimately supporting the integrity of cell division and preventing errors	[358]
*N-*myristoylation& *S-*palmitoylation	Lck	*N-*myristoylation at glycine residue occurs first, followed by post-translational *S-*palmitoylation at cysteine residues	The dual modification promotes Lck's proper localization to the plasma membrane (PM), facilitating interaction with substrates like ZAP70 and PLC-γ1 to initiate TCR signaling	[372]
*N-*myristoylation& *S-*palmitoylation	HGAL	*N-*myristoylation precedes *S-*palmitoylation, and *S-*palmitoylation facilitates HGAL accumulation in lipid rafts	The lipid raft localization enables its interaction with SYK kinase, which increases BCR signaling	[457]
*N-*myristoylation& *S-*palmitoylation	eNOS	*N-*myristoylation is necessary for its membrane association and targeting of the Golgi complex of transfected cells, whereas *S-*palmitoylation influences the targeting of eNOS into caveolae	Localization to caveolae regulates the frequency and magnitude of NO release in response to stimuli in vivo	[80]
*S-*prenylation&*S-*palmitoylation	Ras	S-prenylation at the CAAX motif occurs first, followed by *S-*palmitoylation, which targets Ras to specific membrane microdomains	*S-*prenylation enables effector activation by forming Ras-effector complexes, while *S-*palmitoylation further modulates membrane targeting and functional activation, influencing cellular transformation and phenotypic outcomes	[86]
*S-*prenylation&*S-*palmitoylation	Rac 1	Geranylgeranylation occurs before *S-*palmitoylation, which promotes Rac1's translocation to cholesterol-enriched microdomains within the MAMs after viral infection	MAM-localized Rac1 inhibits MAVS interaction with the E3 ligase Trim31, preventing MAVS ubiquitination, aggregation, and activation	[458]
*S-*prenylation&*S-*palmitoylation	bCdc42	bCdc42 undergoes *S-*prenylation, bypassing proteolysis, and carboxymethylation, and is modified with palmitate at the second cysteine residue	*S-*palmitoylation enriches the bCdc42 on the plasma membrane and enhances its signaling activity	[459]
*S-*prenylation&*S-*palmitoylation	Rap2	Palmitoylation of Rap2 depends on prior *S-*prenylation; it occurs only after *S-*prenylation, targeting Rap2 to recycle endosomes	*S-*palmitoylation ensures Rap2 is targeted to recycling endosomes, where it activates TNIK to regulate cell spreading, adhesion, and morphology	[460]

**Table 2:** The role of *N-*myristoylation in modulating protein functions in the TME

Protein	Functions	References
GRASP	*N-*myristoylation promotes the interaction between GRASP and membrane-associated proteins, facilitating protein transpairing and membrane anchoring	[461]
Src	*N-*myristoylation promotes the dimerization of Src subunits, regulates membrane localization, and participates in signal transduction and intracellular trafficking	[462]
mouse minute virus	*N-*myristoylation facilitates the entry of the nuclear mouse minute virus capsid protein into the nucleus, disrupting membrane structures	[463]
EZH2	*N-*myristoylation of EZH2 facilitates the formation of LLPS droplets, enhancing STAT3 signaling and driving cell proliferation in lung cancer	[180]
NDUFB7, DMAC1	*N-*myristoylation is required for the mitochondrial localization of NDUFB7 and DMAC1 and is critical for the assembly and functionality of mitochondrial complex I and OXPHOS	[15, 136]
AMPK	*N-*myristoylation of AMPK facilitates metabolic reprogramming, promoting glycolysis in cancer cells under metabolic stress, with VPS34 and ATG16 complex components to damage mitochondria, regulating selective mitophagy to maintain cancer cell viability	[221, 222]
ARF1	1. *N-*myristoylation of ARF1 regulates autophagy and STING-dependent autophagy, promoting cell survival under stress 2. ARF1 *N-*myristoylation negatively regulates apoptosis by activating RSK1 and phosphorylating BAD	[223] [253, 254]
AKT	*N-*myristoylation of AKT suppresses autophagy by increasing p62 levels and reducing Beclin- 1 and LC3 expression	[224]
BID	*N-*myristoylation targets the complex of BID p7 and myristoylated p15 fragments to artificial membranes composed of mitochondrial lipids and intact mitochondria. After protein cleavage, *N-*myristoylation acts as an activation switch, enhancing BID-induced cytochrome c release and cell death	[248]
PKA2	*N-*myristoylation promotes the membrane localization of the caspase- 3 cleaved PAK2 C-terminal fragment, enhancing its ability to induce cell death through the c-Jun N-terminal kinase pathway	[249]
DES1	*N-*myristoylation of DES1 targets the mitochondria, affecting mitochondrial sphingolipid metabolism, particularly ceramide production	[200]
FUS1	*N-*myristoylation of FUS1 protein regulates the activities of BAX, and caspase induces cell apoptosis	[255]
FSP1	*N-*myristoylation promotes the membrane localization of FSP1, enhances its inhibitory effect on lipid peroxidation, and regulates ferroptosis	[281]
MARCKS	*N-*myristoylation of MARCKS modifies adhesion capabilities with fibronectin, enhancing cell–matrix interactions	[316]
Rpt2	*N-*myristoylation of Rpt2 alters the membrane-associated proteasome, impairing cell adhesion and membrane protein trafficking	[464]
p22	*N-*myristoylation of p22 facilitates proper folding and binding to microtubules	[319]
FMNL2/3	*N-*myristoylation of FMNL2/3 regulates spindle migration and cell morphology, which is essential for tumor cell movement	[320, 321]
TRAM	*N-*myristoylation of TRAM is critical for its membrane localization, activating NF-κB and IRF3 in response to LP*S-*TLR4 signaling	[326, 327]
Nef	1. *N-*myristoylation of Nef enhances proinflammatory responses by activating MAPK and NF-κB pathways 2. *N-*myristoylation of Nef inhibits dendritic cell endocytosis, upregulating DC-SIGN and promoting T-cell aggregation	[346, 465]

Several NMT inhibitors have been developed, ranging from first-generation broad-spectrum inhibitors to newer selective agents.

ABL001(asciminib) is an allosteric inhibitor that selectively targets the myristoyl pocket of BCR-ABL1, inducing an inactive kinase conformation [22]. Compared to first- and second-generation tyrosine kinase inhibitors, asiminib has fewer off-target effects because of the limited number of tyrosine kinases containing myristate-binding sites. When viability was assessed in a diverse panel of human cancer cell lines, it selectively inhibited the proliferation of BCR::ABL1 expressing leukemic cell lines but showed minimal or no effect on cells not expressing BCR::ABL1 [466]. Recent clinical trials have shown promising antitumor results for asciminib. In a Phase III study (NCT03106779) [467], asciminib achieved a significantly higher major molecular response(MMR) rate and better tolerability compared to the ATP-site inhibitor bosutinib in patients with refractory chronic myeloid leukemia (CML). Additionally, a Phase III trial evaluating asciminib as frontline therapy, which commenced in 2024 (NCT04971226) [468], has also reported encouraging outcomes. Moreover, Phase I results in T315I-mutated chronic-phase CML patients (NCT02081378) revealed that 48.9% of evaluable patients achieved a significant molecular response, with even better responses observed in ponatinib-naïve patients [469]. Importantly, the resistance mutation profile of asciminib does not overlap with that of conventional TKIs, suggesting potential synergistic effects when used in combination therapy [22]. However, mutations in the myristoyl pocket or compensatory downstream pathway activation could drive resistance, necessitating further investigation.

PCLX- 001 (Zelenirstat), the first pan-NMT inhibitor tested in humans, targeting NMT1 and NMT2, interferes with Src family kinases and energy metabolism by promoting the degradation of NDUFAF4, an essential assembly factor for respiratory complex I in cancer cells [470]. Preclinical studies showed that PCLX- 001 induces apoptosis in cancer cells, attributed to loss of Src family proteins *N*-myristoylation and suppression of B-cell receptor survival signaling [446]. Notably, PCLX- 001 exhibited strong activity in hematologic malignancy models (like lymphoma), whereas solid tumor cell lines were somewhat less sensitive, NMT2 suppression may synergistically enhance the therapeutic sensitivity of PCLX- 001 [446]. In vitro treatment with PCLX- 001 significantly reduced viability of BrCa cells and even caused tumor regressions in BrCa xenografts [453], higher doses were needed in solid tumors and dose-limiting weight loss was observed in animal models at those exposures [19]. Phase I clinical trials have demonstrated that Zelenirstat is well tolerated, achieves plasma exposures expected to be efficacious, stable disease as best response was seen in 28% of patients, and exhibits early signs of anticancer activity, thereby supporting its further development [23], but is associated with gastrointestinal toxicity, highlighting the need for improved drug delivery strategies. The broad, “pan-target” mechanism of PCLX- 001 could make it applicable to diverse cancers, but it also raises the risk of on-target toxicity in normal cells that use N-myristoylation, necessitating careful dosing and patient selection.

IMP- 366 and IMP- 1088, newer-generation NMT inhibitors, have shown selective inhibition without off-target cytotoxicity [471], but their clinical potential remains uncertain due to the lack of pharmacokinetic and toxicity data. In vitro studies have demonstrated that IMP- 366 exerts antitumor effects by inducing ferroptosis [472], ER stress, and parthanatos [19]. Some NMT inhibitors have demonstrated promising efficacy in preclinical studies but still encounter challenges, such as off-target effects and delivery limitations. For example, 2-hydroxymyristic acid, B13, and Tris-DBA palladium have shown limited effectiveness in inhibiting *N-*myristoylation and may induce cytotoxicity through NMT-independent mechanisms [471].

### *S*-palmitoylation

*S-*palmitoylation is a dynamic and reversible modification regulated by 23 ZDHHC family members. Here, we list the targets of *S-*palmitoylation in the TME and its associated functions (Table 3) to facilitate a better understanding of its role. Currently, all PATs inhibitors face limitations due to low efficacy or lack of selectivity. Although 2-bromopalmitate (2-BP) has historically been the most commonly used palmitoylation inhibitor, its significant off-target effects and toxicity also render it one of the least ideal inhibitors [473, 474]. In cells, 2-BP is converted into its CoA form, which serves as a substrate for DHHC enzymes, potentially leading to unintended labeling of substrate proteins [473]. Therefore, there is a substantial demand for inhibitors specifically targeting DHHC enzymes. Inhibitors capable of distinguishing between different DHHC enzymes would be even more valuable.

**Table 3:** The role of *S-*palmitoylation in modulating protein functions in the TME

Proteins	Functions	References
CD36	*S-*palmitoylation stabilizes CD36 expression and directs its localization to lipid rafts	[341]
EGFR	*S-*palmitoylation of EGFR activates the PI3 K/AKT pathway and enhances cell proliferation in mutant KRAS lung cancer	[169]
AGK	*S-*palmitoylation of AGK to promote AKT-mTOR activation in renal cell carcinoma	[175]
mTOR	ZDHHC22 decreases mTOR protein stability, reducing activation of the AKT pathway in breast cancer	[176]
TEAD	Auto *S-*palmitoylation of TEAD is critical for binding with YAP/TAZ and the activity of the Hippo signaling pathway	[178, 179]
LDHA	*S-*palmitoylation enhances LDHA activity, promoting glycolysis, lactate production, and TME acidification	[16]
MDH2	*S-*palmitoylation enhances MDH2 catalytic activity, boosting ATP production and supporting tumor cell proliferation	[210]
ULK1	ZDHHC13-mediated *S-*palmitoylation of ULK1 is crucial for autophagy initiation. It enhances ATG14L phosphorylation, which activates PI3-Kinase and produces phosphatidylinositol 3-phosphate, a key lipid in the autophagosome membrane	[227]
Beclin 1	DHHC5-mediated *S-*palmitoylation of Beclin 1 enhances its interaction with ATG14L and VPS15, promoting autophagy	[231]
ATG16L1	*S-*palmitoylation of ATG16L1 promotes its interaction with WIPI2B and Rab33B, ensuring efficient autophagosome formation	[233]
P62	*S-*palmitoylation of p62 increases its affinity for LC3-positive autophagosomes, thus strengthening the selective degradation of ubiquitinated proteins	[238]
DR4	*S-*palmitoylation of DR4 regulates their raft localization and oligomerization	[475]
BAX	*S-*palmitoylation of BAX is necessary for its apoptotic activity by binding to the mitochondrial outer membrane	[266]
neurotensin receptor- 1	*S-*palmitoylation of neurotensin receptor- 1 enhances its interaction with Gαq/11, thus inhibiting apoptosis in breast cancer	[269]
SLC7 A11	*S-*palmitoylation regulates the stability of SLC7 A11 by reducing its ubiquitination, and AMPKα1 can further enhance this interaction in glioblastoma tumors	[286]
GSDMD	ZDHHC5/7/9-mediated *S-*palmitoylation of GSDMD facilitates its activation and pore formation, triggering pyroptosis *S-*palmitoylation is essential for pore formation but does not affect GSDMD cleavage	[293, 294]
NLRP3	ZDHHC1 enhances the interaction between NLRP3 and NEK7, promoting inflammasome assembly ZDHHC5 of NLRP3 increases its degree of membrane binding and stability, further supporting practical inflammasome function ZDHHC12 prevents sustained inflammation by limiting NLRP3 inflammasome activation through chaperone-mediated autophagy	[298–300]
NOD1/2	*S-*palmitoylation of NOD1/2 promotes their membrane recruitment and immune signaling	[342]
cGAS	*S-*palmitoylation of cGAS inhibits its binding to DNA, negatively regulating antitumor immune responses	[331]
STING	*S-*palmitoylation of STING aggregates at the Golgi apparatus and drives IFN*-*I response	[476]
IFNAR1	*S-*palmitoylation of IFNAR1 accelerates degradation, preventing sustained activation and activating STAT2	[335]
CCR5	*S-*palmitoylation of CCR5 plays a critical role in its intracellular trafficking, and the absence of *S-*palmitoylation reduces its human immunodeficiency virus coreceptor function	[336]
FBXO10	*S-*palmitoylation of FBXO10 localizes it to the membrane, promoting HGAL ubiquitination and degradation	[477]
NKG2D	*S-*palmitoylation of NKG2D promotes aggregation with MICA/MICB in cholesterol-rich membrane domains	[351, 352]
Syntaxin 11	*S-*palmitoylation of syntaxin 11 ensures its localization at immune synapses, enhancing NK cell cytotoxicity	[353]
IFNGR1	*S-*palmitoylation of IFNGR1 at Cys122 enables AP3D1-mediated lysosomal degradation, and preventing this process helps maintain the integrity of IFNγ and MHC-I signaling	[427]
CD80	*S-*palmitoylation protects CD80 protein from ubiquitination-mediated degradation, regulates protein stability, and ensures accurate plasma membrane localization	[365]
ZAP70	*S-*palmitoylation of ZAP70 promotes its phosphorylation of LAT and SLP76	[376]
PD-L1	*S-*palmitoylation of PD-L1 stabilizes the protein by preventing monoubiquitination and blocking its degradation through the ESCRT pathway and lysosomal degradation	[21]
PD- 1	*S-*palmitoylation of PD- 1 prevents its degradation, enhances stability, and activates mTOR, promoting tumor proliferation	[138]
TIM- 3	*S-*palmitoylation of TIM- 3 inhibits its interaction with HRD1, preventing ubiquitination and degradation, thereby stabilizing the TIM- 3 protein	[390]
Mpp7	*S-*palmitoylation of Mpp7 regulates the Rap1 signaling pathway, influencing cancer progression	[397]
Wrch- 1	*S-*palmitoylation of Wrch- 1 regulates the membrane localization and promotes fibroblast growth and transformation	[400]
Rho J	*S-*palmitoylation enhances localization and activation of Rho J and promotes angiogenesis and EC motility	[420]
VEGFR1	*S-*palmitoylation of VEGFR1 enhances its stability in the membrane and reduces angiogenic activity	[417]
Estrogen Receptor 46	*S-*palmitoylation of Estrogen Receptor 46 activates the PI3 K-Akt-eNOS pathway	[412]

The high structural similarity and functional redundancy among ZDHHC isoforms present a significant challenge for the development of selective inhibitors. Multiple overlapping ZDHHC enzymes regulate *S*-palmitoylation, complicating the design of isoform-specific inhibitors. Therefore, repositioning drug development strategies is necessary, such as using proteolysis-targeting chimeras (PROTACs)s, which may offer higher specificity for targeting these enzymes, and experimental evidence supports their feasibility [478]. Recent studies have highlighted the important role of ZDHHC20 [110, 169–171, 365] in digestive tract tumorigenesis and metastasis, as well as the role of ZDHHC9 [138, 386, 401] in antitumor immunity and immune checkpoints in the TME. Both ZDHHC9 and ZDHHC20 exhibit characteristics of potential tumor biomarkers, supported by multiple datasets and clinical correlations. In pancreatic cancer (GSE16515 dataset), ZDHHC20 mRNA is overexpressed compared to normal tissues (Fold Change = 1.004) [479], and high ZDHHC20 expression correlates with worse survival in pancreatic [479] and liver cancer patients [110]. In 68 glioblastoma patients, high ZDHHC9 expression and PM-localized GLUT1 are linked to significantly shorter median overall survival (778 and 436 days vs. 1057 and 1380 days in low-expression patients). Multivariate analysis confirms ZDHHC9 as an independent adverse prognostic factor, adjusting for age, sex, and tumor resection status [480]. Pan-cancer analysis across 33 human cancer types reveals ZDHHC9 mRNA upregulation in 20 cancers, and TISIDB data indicates that ZDHHC9 negatively correlates with key immune regulators, further supporting its role in tumor progression [386]. These findings establish ZDHHC9 and ZDHHC20 as potential biomarkers for tumor prognosis and patient risk stratification. However, due to the multi-layered targets of ZDHHC9/20 and its ability to affect various cellular pathways, off-target effects are a concern, necessitating more stringent regulatory considerations.

*S-*palmitoylation-related inhibitors are currently in the research phase and have not yet entered clinical use, except for TEAD inhibitors. Many early PAT inhibitors were pan-inhibitors (e.g., 2-BP [481], tunicamycin [482], and cerulenin [483]) that broadly suppressed multiple ZDHHC isoforms, However, their significant off-target toxicity and lack of specificity limit their therapeutic potential, rendering them more suitable as experimental tools rather than viable drug candidates.

Recent efforts have shifted toward selective PAT inhibitors, such as TTZ- 1/2 (ZDHHC2) [484], cyanomyracrylamide (acts on a variety of ZDHHCs) [485, 486], MY-D- 43 (ZDHHC3/7/20) [487]. However, these compounds cannot yet be considered truly selective inhibitors, as their specificity has not been rigorously validated. The lack of reliable evaluation systems, specific antibodies, and robust verification methods limits the ability to accurately assess their target selectivity. Achieving high specificity remains a significant challenge.

Current research is increasingly focused on specific *S-*palmitoylated substrates such as PD-L1 and TEAD, particularly in cancer immunotherapy. These studies aim to enhance antitumor immune responses and improve treatment efficacy. Research targeting PD-L1 *S-*palmitoylation has focused on three strategies. 1. Novel delivery methods, e.g., poly (lactic-co-glyceric acid) nanoparticles, can induce immunogenic death in tumor models. However, the efficacy and safety of this delivery method still need to be further validated [488, 489]. 2. Competitive short peptide inhibitors, e.g., CPP-S1, have shown some antitumor effects, but their poor stability in mice may lead to insufficient effective concentration and affect efficacy [21], 3.natural compounds (e.g., benzocuproine C [490]), have been used to reduce PD-L1 levels and activate anti-tumor immune responses. While these strategies provide a promising framework for targeting PD-L1 *S-*palmitoylation, none have progressed to clinical trials, emphasizing the need for further optimization in drug delivery, selectivity, and pharmacokinetics.

TEAD contains a targetable central hydrophobic pocket occupied by palmitate due to self-palmitoylation. TEAD-targeting inhibitors have advanced significantly in clinical development, with multiple drugs entering Phase I trials. VT3989 [491] (NCT04665206), a pan-TEAD inhibitor, effectively blocks *S-*palmitoylation and disrupts YAP-TEAD transcriptional activity, demonstrating broad-spectrum efficacy, whereas IK930 [492] (NCT05228015)—targeting only TEAD1—a selective TEAD 1 inhibitor, exhibits lower toxicity but shows reduced antitumor activity. In contrast, inhibitors targeting TEAD1 and TEAD4 (such as SPR1) appear to better balance antitumor efficacy with toxicity [493].

The potential of AI-driven analytical approaches to uncover lipidation networks is becoming increasingly apparent. By leveraging an AI-generated model, ISM6331 [142] was successfully identified as a potent TEAD inhibitor, demonstrating its reversible binding to TEAD *S-*palmitoylation sites and strong suppression of TEAD transcriptional activity. Moreover, it has received orphan drug designation and entered Phase I clinical trials (NCT06566079), with the first patient dosed in January 2025, though its efficacy remains to be observed.

### *S*-prenylation

Currently, the role of *S-*prenylation in the TME is becoming increasingly clear and important. The targets of *S-*prenylation in the TME are listed in Table 4 to provide a clearer understanding of its role. In recent years, research on FTIs and GGTIs has expanded across various cancer models, with several advancing to clinical trials. FTIs are among the most extensively studied and clinically tested.

**Table 4:** The role of *S-*prenylation in modulating protein functions in the TME

Protein	Functions	References
Rab	Geranylgeranyltransferase regulates Rab1 and Rab11 localization, affecting the trafficking of Scabrous and Delta, thereby modulating Notch signaling	[494]
Ykt6	Farnesylation of the Ykt6 increases its stability and helical folding	[73]
Rheb	S-prenylation of Rheb affects its localization, directing it to intracellular membranes	[173]
CENP-F	Inhibition of farnesylation leads to the loss of Cenp-F in the centromere, which is of significant relevance in breast cancer	[187]
CENP-E	Inhibition of CENP-E farnesylation results in the alteration of the microtubule-centromere interaction during mitosis and results in the accumulation of cells prior to metaphase	[188]
PRL- 3	S-prenylation of PRL- 3 enhances its membrane binding and accelerates autophagy by activating the PIK3 C3-Beclin- 1 complex	[240]
Rap1	S-prenylation of Rap1 is essential for its membrane localization and function in promoting cell–cell adhesion	[495]
ZAP	Farnesylation of ZAP is essential for its targeting of endolysosomes, enhancing antiviral activity	[339]
CRACR2 A	S-prenylation of CRACR2 A ensures its localization to the Golgi, supporting TCR signaling and immune synapse formation	[384]
HIF1α	Inhibition of farnesylation reduces angiogenesis in NSCLC and HNSCC cells by decreasing HIF1α expression and VEGF production	[418]

In earlier studies, it was observed that when farnesylation is inhibited, proteins that are substrates for both FTase and GGTase I, such as KRAS and NRAS, can undergo compensatory geranylgeranylation [45, 48, 49]. This alternative modification allows these proteins to maintain membrane association and function despite preventing farnesylation. Research has shown that the effectiveness of FTIs in certain cancers, such as osteosarcoma [496] and colon carcinoma [497], is limited. In contrast to KRAS and NRAS, HRAS undergoes exclusively farnesylation through FTase, making FTIs potentially helpful in treating HRAS*-*mutant cancers. Therefore, current FTI research primarily focuses on tumors with HRAS mutations or overexpression [498, 499]. For proteins that are substrates for both FTase and GGTase-I, dual inhibiting these enzymes may be an effective strategy for treating tumors and overcoming resistance. Notably, the simultaneous inhibition of FTase and GGTase-I has significantly reduced lung and KRAS*-*driven pancreatic tumors [500, 501].

Since the 1990 s, they have been investigated as potential treatments for HRAS*-*mutant tumors, leading to multiple Phase I–III trials. Compared to GGTIs, PDEδ inhibitors, and Rce1/ICMT inhibitors, FTIs have made the most clinical progress, with some showing promise for specific indications.

Tipifarnib is a FTI that, while nominally targeting FTase, broadly affects all *S-*prenylation of proteins requiring farnesyl (most famously RAS). Accordingly, Tipifarnib has found a niche in cancers with HRAS mutations. In a recent phase II trial for recurrent HRAS-mutant HNSCC, tipifarnib achieved a 55% objective response rate(ORR) in patients with high mutant allele frequency, with median progression-free survival ~ 5.6 months on tipifarnib versus 3.6 months on prior therapy^488^. It also get an ORR of 39.7% in peripheral T-cell lymphoma (PTCL) patients in a Phase II open-label trial (NCT02464228) [504]. These results led to FDA breakthrough therapy designation for HRAS-driven tumors. Still, tipifarnib’s efficacy is contingent on tumor genotype and TME which works best in high HRAS output patients and is ineffective against KRAS-driven cancers unless combined with a geranylgeranyl inhibitor [500] or KRAS-G12 C inhibitor [505]. This underscores a key point in selective vs. pan-targeting: a narrower inhibitor (asciminib or tipifarnib) can be very effective in the right molecular setting with fewer side effects, while a broader inhibitor (PCLX- 001) might treat a wider range of tumors but risks lower therapeutic index if normal cells utilize the same lipidation pathways.

Lonafarnib is an FDA-approved drug that specifically inhibits farnesyltransferase, blocking the farnesylation of progerin protein to treat progeria [506]. It has been shown to enhance RAS/MAPK pathway inhibition in combination with anaplastic lymphoma kinase-tyrosine kinase inhibitor in a neuroblastoma model [507]. However, not all clinical trials of FTIs have been successful. The phase III trial (NCT00109538) for CML has been terminated, and the exact results are not yet available. The high frequency of NRAS and KRAS mutations in CML and the compensatory activation of post-geranylgeranyl geranylgeranylation may lead to poor efficacy.

This underscores the heterogeneous response of different malignancies to FTIs, emphasizing the need for precision medicine approaches to identify responsive patient subsets. A next-generation FTI, KO- 2806, has entered a Phase I clinical trial (NCT06026410) and has demonstrated enhanced anti-angiogenic effects when combined with Cabozantinib, a multi-targeted tyrosine kinase inhibitor, primarily by inhibiting RHEB farnesylation in precise cell renal cell carcinoma models [496, 497, 508]

Concerning GGTIs, GGTI- 2418 (PTX- 100) has entered a Phase I clinical trial (NCT03900442), and preliminary data show that it has some anticancer activity in T-cell lymphomas [509]. PTX- 100 achieved an ORR of 40% and a disease control rate (DCR) of 60% in TCL patients, particularly within the cutaneous T-cell lymphoma (CTCL) subgroup. However, its efficacy in solid tumors remains limited, likely due to rapid metabolic clearance leading to suboptimal drug concentrations [510]. The combined inhibition of FTIs and GGTIs has been explored to overcome resistance mechanisms.

In a KRAS-G12D-induced lung cancer model, dual inhibition significantly delayed tumor progression [500]. Further toxicity evaluation is required for its clinical application. PDEδ is a molecular chaperone for farnesylated KRAS and RHEB, crucial in oncogenic KRAS signaling and mTORC1 activation. PDEδ inhibitors have emerged as a potential approach to disrupt KRAS-driven oncogenesis. PDEδ inhibitors (e.g., Deltasonamide1 [174] and NHTD [511]) block the KRAS and mTORC1 signaling pathways by interfering with the membrane localization of KRAS and Rheb. Research has combined PROTAC-based PDEδ degradation with drug development, demonstrating effective inhibition in the MVA pathway and providing promising lead compounds for the development of KRAS mutation-targeted therapies [512, 513].

Rce1 and ICMT inhibitors target post-prenylation modifications, representing another potential avenue for disrupting RAS-driven tumorigenesis. Rce1 inhibitors delay RAS membrane localization, enhancing FTI efficacy [514]. Meanwhile, ICMT inhibitors impede mTOR-mediated autophagy, disrupt tumor cell cycle progression, and augment anticancer activity [515–518].UCM- 1336 is a new generation of ICMT inhibitors, a promising drug candidate with more potent enzyme inhibition and lower toxicity than cysmethynil [517, 519]. However, these inhibitors are still in preclinical studies and require further pharmacokinetic and clinical feasibility studies.

Statins and bisphosphonates have demonstrated antitumor potential by targeting the MVA and *S-*prenylation pathways. Bisphosphonates, such as alendronate-like 3-PEHPC, specifically inhibit GGTase II, thereby blocking *S-*prenylation and reducing tumor burden in bone and the TME [520–524]. Statins, conversely, inhibit autophagy, oncogenic signaling, and inflammation while enhancing endothelial function [272, 525–528]. However, the effects of statins on certain cancers remain complex, as compensatory activation of Rho GTPase may increase aggressiveness and alter tumor cell plasticity [529], necessitating further research into the optimal therapeutic setting.

### Comprehensive comparasion

Inhibitors like ABL001, Tipifarnib, and TEAD inhibitors have shown promising results in clinical trials, albeit with different levels of efficacy and selectivity across various cancer types. Selective inhibitors, such as ABL001, typically demonstrate higher efficacy with fewer off-target effects [466], whereas pan-inhibitors like PCXL- 001 exhibit broader activity but are associated with increased toxicity [446]. For example, ABL001 achieved a 48.9% MMR in T315I-mutated chronic-phase CML patients [469]. while PCXL- 001 resulted in stable disease as the best response in 28% of patients with advanced solid tumors and relapsed/refractory B-cell lymphomas [23]. Similarly, Tipifarnib demonstrated a 39.7% ORR in HRAS-mutated and overexpressed tumors, including peripheral T-cell lymphoma [504]. This highlights the variability in efficacy across different types of cancers, even within the same class of drugs.

In the case of TEAD inhibitors, the pan-TEAD inhibitor VT3989 shows broad treatment potential but comes with significant off-target effects which achieved partial responses in 14.3% of mesothelioma patients but also caused significant adverse effects, including proteinuria, liver enzyme elevations, and cardiomyopathy [491]. In contrast, IK930, a selective TEAD1 inhibitor, demonstrated a more favorable safety profile [492, 493]. This suggests that selective inhibitors may provide similar efficacy with fewer side effects, making them a promising alternative for targeted therapy.

### Resistance mechanisms

RAS-driven tumors exemplify how bypass pathways limit lipidation-targeted therapies. In KRAS- or NRAS-mutant cancers, FTIs failed because the tumor simply utilized geranylgeranyltransferase to prenylate RAS instead [496, 502, 503]. Modern approaches address this by either combining inhibitors (dual FTase/GGTase inhibition) or by patient selection (targeting HRAS mutants that lack an alternate *S-*prenylation route). Likewise, tumors could develop resistance to pan-lipidation inhibitors by upregulating parallel pathways or tolerating loss of the modified protein. Recent findings in a KRAS-driven lung cancer model suggest that loss of *N*-myristoylation is particularly catastrophic when certain mitochondrial import factors TIM17 A are high, whereas cells without that dependency cope better [19]. Such insights hint that vulnerabilities to pan-inhibitors may vary by context, and resistance may involve adaptive rewiring of signaling or metabolism. Even highly selective drugs face resistance; asciminib, for example, can select for BCR-ABL1 mutants outside the myristate pocket that restore kinase activity or prevent drug binding.

S-palmitoylation-related resistance mechanisms add another layer of complexity. As PATs share high structural homology, tumors may evade inhibition by compensating with alternative PATs. As PATs share high structural homology and the redundancy within the ZDHHC family, where different enzymes can catalyze the *S-*palmitoylation of the same substrate, such as more than half of the ZDHHCs were capable of increasing IFITM3 *S-*palmitoylation [530] or the intracellular S0-S1 loop of BK channels is controlled by ZDHHC22 and ZDHHC23 [531]. This redundancy provides cells with flexibility and fault tolerance, it complicates therapeutic targeting. Moreover, APTs provide another escape mechanism by accelerating depalmitoylation, counteracting the effects of PAT inhibitors. For instance, APT1 and APT2 can rapidly depalmitoylate substrates like Ras, restoring their cytosolic localization and bypassing PAT blockade [88].Developing selective inhibitors for specific isoforms like ZDHHC9 or ZDHHC20, which are linked to tumor progression and immune checkpoint regulation, is particularly challenging due to compensatory mechanisms within the ZDHHC network.

Overall, a balance must be struck: selective lipidation-targeted drugs offer precision but need the right biomarkers (e.g. ABL1 mutation or HRAS status), whereas pan-targeting drugs can hit multiple oncogenic pathways at once but may require innovative delivery or combinations to avoid toxicity and overcome tumor adaptability.

### Delivery challenge

The current mainstream delivery methods for lipidation-targeting drugs can be divided into three major strategies: Nanoparticle-Based Delivery, Antibody–Drug Conjugates (ADCs), and PROTACs.

#### Nanoparticle-based delivery

Advanced drug delivery systems are being explored to improve the therapeutic index of lipidation inhibitors. Nanoparticle formulations, such as liposomes, polymeric nanoparticles, or lipid nanocarriers, can preferentially deliver these drugs to tumors, enhancing efficacy while reducing systemic exposur [532]. A striking example is the use of liposomes to co-deliver *S-*prenylation inhibitors in RAS-driven cancers, researchers encapsulated a GGTI in pH-sensitive liposomes and combined it with a FTI [533], this design takes advantage of two key characteristics of the TME: the acidic conditions resulting from hypoxia and the low pH found in intracellular lysosomes [534]. By leveraging the acidic TME, the liposome-encapsulated GGTI is preferentially released in tumor regions, blocking both geranylgeranylation and farnesylation while sparing healthy tissues from toxicity. Notably, 28 μg/ml of liposome-GGTI combined with 2.5 μM FTI suppressed cell proliferation by approximately 80% in KRAS mutant pancreatic cancer cells—an effect neither agent achieved on its own. The resulting combination index of 0.5102 confirms a synergistic interaction, effectively silencing ERK signaling in these RAS-driven tumors [533].

Nanoparticle delivery of lipidation-targeting drugs offers key advantages by improving pharmacokinetics, protecting agents like tipifarnib from rapid metabolism, and allowing tissue-specific targeting. For example, brain-permeable nanocarriers [535] may allow FTase or *S-*palmitoylation inhibitors to cross the blood–brain barrier for treating CNS tumors. Moreover, the enhanced permeability and retention (EPR) effect in the tumor microenvironment enables higher local concentrations of inhibitors, reducing systemic toxicity—particularly important for pan-inhibitors such as PCLX- 001.Nonetheless, challenges remain: nanoparticle accumulation in the brain may cause neurotoxicity, and the high production costs and complex manufacturing limit large-scale use and clinical adoption. Further research is thus essential to refine and simplify nanoparticle formulations for broader application.

#### ADCs

ADCs represent another targeted delivery strategy being applied to lipidation inhibitors. The concept is to use a tumor-specific antibody as a “guided missile” to carry a potent inhibitor directly into cancer cells [536]. Traditionally, ADC payloads are cytotoxic chemotherapies, but recent innovations have expanded payloads to novel mechanism agents. Notably, Myricx Bio is developing NMTi-armed ADCs, where an NMT inhibitor is chemically tethered to an antibody targeting a tumor antigen [537]. A Trastuzumab-NMTi ADC demonstrated potent, selective anti-tumor activity in preclinical models. In vitro, it showed an EC₅₀ of 0.2 nM in HER2⁺ BT474 cells and remained inactive in HER2⁻ MCF7 cells at concentrations up to 100 nM. This approach concentrates a pan-NMT inhibitor in tumor cells, resulting in robust tumor regression while maintaining a favorable safety profile and shows minimal off-target effects.This strategy combines the specificity of biologics with the robust intracellular activity of small molecules, potentially overcoming the distribution challenges faced by free drugs.

#### PROTACs

PROTACs have emerged as a powerful modality for targeting “undruggable” oncoproteins by harnessing the cell’s ubiquitin–proteasome system to induce degradation rather than inhibition. This approach is particularly impactful for lipidated proteins, such as KRAS, whose complete removal is advantageous regardless of mutation status. For instance, the KRAS G12 C–targeting PROTAC LC‑2 links a KRAS G12 C–binding moiety (from a covalent inhibitor) with a VHL ligand, inducing rapid, sustained degradation of endogenous KRAS G12 C with DC50 values ranging from 0.25 to 0.76 μM, which suppresses downstream MAPK signaling [538]. Building on this, Popow et al. broadened the approach to target multiple KRAS mutations by employing noncovalent ligands for both KRAS and VHL—an optimization guided by co‑crystal structures of the KRAS:PROTAC:VHL ternary complex. Their optimized PROTAC selectively degraded 13 of the 17 most common KRAS mutants, proving more effective than inhibition in driving tumor regression in experimental models. Through iterative drug design, they developed ACBI3, which demonstrated excellent pharmacokinetic properties in mice. When administered intravenously, subcutaneously, or intraperitoneally, ACBI3 reached plasma concentrations that exceeded the predicted in vivo DC50 of approximately 281 nM and maintained effective levels for over six hours. In the GP2 d xenograft model, daily dosing at 30 mg/kg significantly reduced intratumoral KRASG12D levels to 44% of control and achieved a remarkable 127% tumor growth inhibition over a 14‑day period—leading to marked tumor regression without significant changes in body weight [539]. Importantly, given that KRAS is a prenylated (farnesylated) protein, this strategy not only removes a lipidated oncoprotein from the cell but also inspires similar approaches—such as PROTAC degraders of the KRAS activator SOS1 [540] and the integration of PDEδ inhibitors that disrupt KRAS membrane localization to effectively target KRAS mutations [512, 513].

PROTACs in lipidation therapeutics can irreversibly knock down key cancer drivers that depend on membrane localization, such as RAS and SRC, rather than merely transiently inhibiting them. This approach may prevent tumor cells from reactivating the same pathway through mutations or compensatory loops by converting difficult targets into degradable ones. Although no PROTAC drug candidate has yet reached approval for a lipidated oncoprotein target and further optimization is underway, early results are encouraging. Delivery challenges for these larger bifunctional molecules remain, but research on nanoparticle-based delivery systems [541, 542] and antibody-PROTAC conjugates is advancing [543]. Overall, PROTAC technology complements traditional lipidation inhibitors by offering a way to target the protein substrate rather than the lipidation enzyme which potentially broadening the scope of lipidation-targeted therapy.

## Perspectives

Lipidation is rapidly emerging as a key regulatory mechanism within the TME. Despite growing interest in this PTM many aspects of its function within the TME remain poorly understood, particularly regarding its role in inflammatory cells, immune cells, and the extracellular matrix. Advances in dynamic lipidation capture, AI-driven discovery, therapeutic targeting, and multi-omics integration are expected to drive the next wave of research and clinical applications. Below, we highlight critical areas for future exploration and innovation.

One of the major challenges in lipidation research is the difficulty in capturing dynamic lipidation events and understanding their spatial heterogeneity within tumors. The emergence of single-cell proteomics [544] and spatial proteomics [545] provides a promising solution to these challenges. Techniques such as spatially resolved MALDI-MSI [114] and NanoSIMS [546] allow for the visualization of lipidated proteins across distinct cellular compartments within the TME, revealing how lipidation patterns change in response to environmental factors such as hypoxia, immune infiltration, or metabolic stress.

Lipidation-driven phase separation is a key regulator of protein organization and signaling in the TME, facilitating the formation of membraneless condensates that amplify oncogenic pathways and suppress immune responses. By enhancing protein hydrophobicity, lipidation promotes the dynamic compartmentalization of signaling molecules into liquid-like structures. This process enables efficient signal transduction and cellular regulation [369], as seen in palmitoylated Ras isoforms forming "rasosomes" [547] for sustained MAPK activation and myristoylated EZH2 driving epigenetic reprogramming [180]. The biophysical properties of these condensates—viscosity, elasticity, and permeability—are modulated by lipidation, influencing protein stability and signal amplification. Ultimately, lipidation-mediated phase separation acts as a molecular scaffold, controlling the spatial and temporal dynamics of tumor progression and immune modulation in the TME.

AI is poised to transform lipidation research by enabling functional predictions of how lipidation affects protein behavior in the TME. Future AI-driven approaches will integrate deep-learning molecular dynamics to model lipidation’s impact on protein conformation, phase separation, and interaction networks. AI-powered virtual screening could accelerate the discovery of inhibitors targeting key lipidation enzymes like ZDHHCs, NMTs, and prenyltransferases. Additionally, AI-based multi-omics analysis will identify lipidation biomarkers linked to tumor progression, immune evasion, and therapy resistance, advancing patient stratification and precision medicine.

Targeting lipidation-dependent pathways represents a novel therapeutic strategy for reprogramming the TME. Several lipidation inhibitors have already entered clinical development, with TEAD *S-*palmitoylation inhibitors ISM6331 [142] and NMTi PCLX- 001 [446], FTI Tipifarnib [502]. Beyond these emerging inhibitors, the development of lipid-mimetic drugs could provide a new class of therapeutics. For instance, interfere with PD-L1 *S-*palmitoylation could enhance immune checkpoint blockade therapy, preventing tumors from suppressing T-cell activation [21]. Similarly, lipidation-based PROTACs could be designed to selectively degrade oncogenic lipidated proteins [538]. Additionally, lipidation-targeted therapies may improve CAR-T cell efficacy [390], as engineering lipid-modified CAR-T cells could enhance their persistence, trafficking, and function in lipid-rich TMEs.

integrating multiomics into large-scale tumor classification efforts could reveal how lipidation patterns correlate with tumor mutational burden, metabolic states, and immune phenotypes, ultimately guiding personalized therapy decisions.

Beyond cancer, the role of lipidation extends to aging [548], immune regulation [549], and neurodegenerative diseases [550], suggesting potential common therapeutic strategies for both cancer immunotherapy and neuroimmune modulation. Exploring these connections may open new avenues for treating age-related diseases and inflammation-driven cancers.

Lipidation is increasingly recognized as a central regulator of the TME, with broad implications for oncogenic signaling, immune suppression, metabolic adaptation, and therapeutic resistance. Moving forward, research should focus on: (1) mapping lipidation dynamics using single-cell and spatial lipidomics; (2) leveraging AI for functional lipidation prediction and drug discovery; (3) developing next-generation lipidation inhibitors for cancer immunotherapy; (4) integrating lipidomics with other omics layers to redefine TME classification; and (5) exploring lipidation’s broader role in aging and neuroimmune interactions. By addressing these key challenges, lipidation research will not only deepen our understanding of the TME but also unlock novel therapeutic strategies to reprogram the tumor microenvironment and improve patient outcomes.

## Conclusions

In summary, lipidation influences nearly all aspects of tumor cells and the surrounding environment, and targeting related lipidation enzymes halts tumor development. However, drugs capable of explicitly targeting these enzymes are currently lacking since lipidation often has redundant modifications that make it challenging to accomplish antitumor tasks for monospecific targets. Encouragingly, some of these inhibitors have advanced to clinical trials or received approval for clinical use, offering hope for the potential cure of certain cancer patients and providing clinicians with more effective treatment options. Continued efforts to refine the specificity and safety of lipidation inhibitors, coupled with a deeper understanding of their molecular mechanisms in the TME, hold the potential to overcome current limitations.

## Data Availability

No datasets were generated or analysed during the current study.

## Abbreviations

ABHD: Α/β Hydrolase domain
ACSL4: Acetyl-CoA synthetase long-chain family member 4
acyl-RAC: Acyl-resin assisted capture
ADCs: Antibody–Drug Conjugates
AI: Artificial Intelligence
AKT: Protein kinase B
AMPK: AMP-activated protein kinase
APT: Acyl protein thioesterase
AR: Androgens receptor
ARF1: ADP-ribosylation factor 1
ASC: Speck-like protein
ATP: Denosine triphosphate
BAX: Bcl- 2-associated X
BCR: B cell receptor
BrCa: Breast cancer
Bregs: Regulatory B cells
BTK: Bruton’s tyrosine kinase
CAAX: C-terminal Cysteine-Aliphatic-Aliphatic-X
CAF: Cancer-associated fibroblast
CaM: Calmodulin
CDC42: Cell division control protein
CDK2: Cyclin-dependent kinase 2
cGAS: Cyclic GMP-AMP synthase
CLMB: Calcimembrin
CoQ₁₀: Coenzyme Q₁₀
CPTAC: Clinical Proteomic Tumor Analysis Consortium
ctBID: C-terminal BID
ctPKA2: C-terminal p21-activated protein kinase 2
DAMP: Damage-associated molecular patterns
DCs: Dendritic cells
DES1: Desulfhydrase 1
DR4: Death Receptor 4
EC: Endothelial cells
ECM: Extracellular matrix
EGFR: Epidermal growth factor receptor
EMT: Epithelial–mesenchymal transition
eNOS: Endothelial nitric oxide synthase
EPR: Enhanced permeability and retention
ER: Endoplasmic reticulum
ERK: Extracellular signal-related kinases
ESCRT: Endosomal sorting complex required for transport
eTreg: Effector Treg
EZH2: Enhancer of zeste homolog 2
FASN: Fatty acid synthase
FGF: Fibroblast growth factor/
FGFR: FGF receptor
FPP: Farnesyl pyrophosphate
FRET: Förster resonance energy transfer
FRS2α: Fibroblast growth factor receptor substrate
FSP1: Ferroptosis suppressor protein 1
FTase: Farnesyltransferase
FTIs: FTase inhibitors
GDIs: Guanine nucleotide dissociation inhibitors
GGPP: Geranylgeranyl pyrophosphate
GGPS1: Geranylgeranyl pyrophosphate synthase 1
GGTase: Geranylgeranyltransferase
GPR81: G-protein coupled receptor 81
GPX4: Glutathione peroxidase 4
GRASP: Golgi reassembly stacking protein
GSDMD: Gasdermin D
GSDMD-NT: GSDMD N-terminal domain
HGAL: Human germinal center-associated protein
HGF: Hepatocyte growth factor
HIF- 1α: Hypoxia-inducible factor 1-alpha
HLA: Human leucocyte antigen
HNSCC: Head and neck squamous cell carcinoma
HO- 2: Heme oxygenase- 2
HsNMT1: Homo sapiens NMT1
ICMT: Isoprenylcysteine carboxyl methyltransferase
IEDDA: Inverse electron-demand Diels–Alder
IFN: Interferon
IFNAR1: IFN*-*alpha receptor subunit 1
IFN*-*I: Type I IFN
IL: Interleukin
IRF3: IFN regulatory factor3
ITAM: Immunoreceptor tyrosine-based activation motif
JNK: C-Jun N-terminal kinase
LAT: Linker for activation of T cell
LC–MS/MS: Liquid chromatography-tandem mass spectrometry
LDHA: Lactate dehydrogenase A
LLPS: Liquid–liquid phase separation
LPS: Lipopolysaccharide
MAL: MyD88 adapter-like
MALDI-MSI: Matrix-assisted laser desorption/ionization mass spectrometry imaging
MAM: Mitochondria-associated membrane
MAPK: Mitogen-activated protein kinase
MARCKS: Myristoylated alanine-rich C-kinase substrate
MCT4: Monocarboxylate transporter 4
MCT1: Monocarboxylate transporter 1
MDH2: Malate dehydrogenase 2
MET: Mesenchymal–epithelial transition
Met AP2: Methionine aminopeptidase 2
MHC: Major histocompatibility complex class
MIF: Migration inhibitory factor
MMP- 2: Matrix metalloproteinases 2
MMP- 9: Matrix metalloproteinases 9
MMR: Major molecular response
Mpp6: MAGUK p55 subfamily member 6
Mpp7: MAGUK p55 subfamily member 7
MS: Mass spectrometry
mTOR: Mammalian target of rapamycin
mTORC1: Mechanistic target of rapamycin complex 1
MVA: Mevalonate
MyD88: Myeloid differentiation primary response 88
NFATsc: Nuclear factors of activated T cells
NF-κB: Nuclear factor kappa B
NMT: *N-*Myristoyltransferase
NO: Nitric oxide
NOD1/2: Nucleotide-binding oligomerization domain 1/2
OSCC: Oral squamous cell carcinoma
OXPHOS: Oxidative phosphorylation
PA: Palmitic acid
PAMP: Pathogen-associated molecular patterns
PCa: Prostate cancer
PDAC: Pancreatic ductal adenocarcinoma
pDCs: Plasmacytoid dendritic cells
PDE6δ: Phosphodiesterase 6 delta subunit
PI3 K: Phosphoinositide 3-kinase
PKCε: Protein kinase C ε
PLCγ: Phospholipase Cγ
PM: Plasma membrane
PPT1: Palmitoyl protein thioesterase 1
PROTAC: Proteolysis-targeting chimeras
PRR: Pattern recognition receptors
PTM: Post-translational modification
Rce1: Ras converting enzyme 1
REP: Rab escort protein
ROS: Reactive oxygen species
RTKs: Receptor tyrosine kinases
RZZ complex: ROD–Zwilch–ZW10 complex
S6 K: S6 kinase
SFKs: Src-family kinases
SILAC: Stable isotope labeling by amino acids in cell culture
SPAAC: Strain-promoted azide-alkyne cycloaddition
SRS: Stimulated Raman scattering
STAT3: Signal transducer and activator of transcription 3
STING: Stimulator of interferon genes
SYK: Spleen tyrosine kinase
TAMs: Tumor-Associated Macrophages
TAZ: Transcriptional coactivator with PDZ-binding motif
TCR: T cell receptor
TEAD: TEA domain family member
TIM- 3: T-cell immunoglobulin and mucin domain-containing protein 3
TLR4: Toll-like receptor 4
TME: Tumor microenvironment
TNF: Tumor necrosis factor
TRAM: Trif-related adapter molecule
Treg: Regulatory T
Trif: Toll/IL- 1 receptor domain-containing adapter inducing IFN*-*β
UAAs: Unnatural amino acids
ULK1: Unc- 51-like kinase 1
UPR: Unfolded protein response
YAP: Yes-associated protein
ZAP70: Zeta-chain associated protein
ZDHHC: Zinc finger DHHC-type
